# Potent anti-influenza H7 human monoclonal antibody induces separation of hemagglutinin receptor-binding head domains

**DOI:** 10.1371/journal.pbio.3000139

**Published:** 2019-02-04

**Authors:** Hannah L. Turner, Jesper Pallesen, Shanshan Lang, Sandhya Bangaru, Sarah Urata, Sheng Li, Christopher A. Cottrell, Charles A. Bowman, James E. Crowe, Ian A. Wilson, Andrew B. Ward

**Affiliations:** 1 Department of Integrative Structural and Computational Biology, The Scripps Research Institute, La Jolla, California, United States of America; 2 Department of Pathology, Microbiology and Immunology, Vanderbilt University School of Medicine, Nashville, Tennessee, United States of America; 3 Department of Medicine, University of California San Diego, La Jolla, California, United States of America; 4 Vanderbilt Vaccine Center, Vanderbilt University Medical Center, Nashville, Tennessee, United States of America; 5 Department of Pediatrics, Vanderbilt University Medical Center, Nashville, Tennessee, United States of America; 6 The Skaggs Institute for Chemical Biology, The Scripps Research Institute, La Jolla, California, United States of America; University of Pittsburgh, UNITED STATES

## Abstract

Seasonal influenza virus infections can cause significant morbidity and mortality, but the threat from the emergence of a new pandemic influenza strain might have potentially even more devastating consequences. As such, there is intense interest in isolating and characterizing potent neutralizing antibodies that target the hemagglutinin (HA) viral surface glycoprotein. Here, we use cryo-electron microscopy (cryoEM) to decipher the mechanism of action of a potent HA head-directed monoclonal antibody (mAb) bound to an influenza H7 HA. The epitope of the antibody is not solvent accessible in the compact, prefusion conformation that typifies all HA structures to date. Instead, the antibody binds between HA head protomers to an epitope that must be partly or transiently exposed in the prefusion conformation. The “breathing” of the HA protomers is implied by the exposure of this epitope, which is consistent with metastability of class I fusion proteins. This structure likely therefore represents an early structural intermediate in the viral fusion process. Understanding the extent of transient exposure of conserved neutralizing epitopes also may lead to new opportunities to combat influenza that have not been appreciated previously.

## Abstract 2

Cryo-EM reveals a new epitope between influenza virus hemagglutinin head protomers that is only transiently exposed, yet leads to a potent, pan H7 influenza-neutralizing response; this unique mechanism of action represents a potential new opportunity to combat influenza.

## Author summary

Influenza viruses cause severe respiratory infections on a global scale annually. Vaccine efforts are hampered by the virus’s naturally high mutation rate, which results in wide variation between influenza strains of the antigens that are produced and recognized by antibodies, particularly in the surface glycoprotein hemagglutinin (HA). However, broadly neutralizing antibodies (bnAbs) are a class of antibodies that develop during natural infections that are capable of inhibiting infection across multiple strains. In this study, we structurally characterized one such bnAb, H7.5, which targets a unique semioccluded yet highly conserved region on the HA head. We showed, using both negative-stain and high-resolution cryo-electron microscopy (cryoEM), that after a short incubation, H7.5 fragment antigen binding (Fab) induces HA to fall apart, effectively preventing infection. We found that H7.5 binds to an epitope only accessible through transient “breathing” of the HA head, and this observation provides insight into the conformational transitions necessary for viral fusion as well as key information about a unique vaccine target.

## Introduction

Influenza displays three glycoproteins that embroider the viral surface: hemagglutinin (HA), neuraminidase (NA), and Matrix-2 ion channel. All of these proteins are necessary for the viral replication cycle. Among the surface glycoproteins, HA is the principal target for neutralizing antibodies. HA is a class I viral fusion protein that facilitates viral entry by interacting with sialic acid receptors on the host cell and then fusing the viral and cell membranes in acidic endosomal compartments. HA is expressed as a precursor form termed HA0, which is then cleaved by host-cell proteases into HA1 and HA2 domains, resulting in a trimer of heterodimers. HA1 contains the membrane-distal sialic acid receptor-binding site (RBS), while HA2 includes the fusion machinery, proximal to the membrane. Proteolytic cleavage at the HA1–HA2 junction liberates the hydrophobic fusion peptide, which then becomes buried in the center of the trimer. The HA1 and HA2 domains remain covalently linked by a disulfide bond after cleavage. This cleaved, prefusion conformation is metastable and poised to undergo pH-induced conformational changes but must not do so prematurely.

After influenza virus binds to the host cell, it is endocytosed and trafficked into endosomal compartments, in which the lumen is acidified. Near pH 5.5, HA undergoes large conformational rearrangements, which lead to insertion of its fusion peptide into the host membrane. This process drives fusion of the host and viral membranes and release of the viral RNA genome into the cytoplasm [1].

Influenza evolution, particularly in HA, occurs rapidly, with antigenic drift sometimes conferred by single amino acid changes near the RBS [2]. The RBS, which is the most conserved region of the HA head, forms a shallow pocket to which a series of antibodies has been shown to bind and neutralize [3]. Although amino acid mutations in HA are used to evade the host immune system, there are conserved areas of HA that are vital to viral fitness. Head-binding antibodies have been shown to be very effective in neutralizing the virus but usually only in a strain-specific manner, although some broadly neutralizing antibodies are known to target the RBS [3,4]. Monoclonal antibody (mAb) 5J8, a prototypic head antibody with breadth, targets the RBS via a long complementary determining region heavy chain 3 (CDRH3) that mimics the sialoglycan receptor and neutralizes H1 strains from 2009 to the pandemic of 1918 [5]. RBS antibodies typically have a broader neutralization profile due to sequence conservation of this site, while other regions on the HA1 head are hot spots for strain-specific antibodies [6].

Originating as avian influenza, H7 strains have crossed over intermittently to infect humans [7]. Laboratory studies have shown only three amino acids are required to completely change receptor specificity from avian to human, allowing human-to-human transmission and increasing the chance of a new pandemic [8]. In fact, an outbreak of H7N9 virus in China in 2013 has been linked to close contact of humans with chickens and ducks, which were identified as a reservoir for the virus [7,9]. Since then, H7N9 has continued to circulate in poultry reservoirs, causing a spike in human infections in recent years [7]. Several H7-specific mAbs have been described recently, including H7.137, H7.167, and H7.169, which target highly conserved regions of the HA head adjacent to the RBS [10]. Another antibody isolated in the same study, mAb H7.5, was shown to recognize an epitope that overlaps a portion of the H7.167 epitope and neutralize similar strains of human H7 virus, including those from mallards and chickens, but which binds at a different angle [10]. The H7.5 mAb potently neutralizes human H7 strains of influenza virus, including strains isolated in outbreaks from 2003–2013 in Shanghai, the Netherlands, and New York [10].

In the course of our epitope-mapping studies of H7-specific mAbs using negative-stain electron microscopy (nsEM), we noted a striking effect of mAb H7.5 binding to HA, in which the soluble H7 ectodomain trimer falls apart. Using biochemical and structural approaches described here, we delineate the phenotypic effect of H7.5 on HA trimers. We also report several cryo-electron microscopy (cryoEM) structures of H7.5 fragment antigen binding (Fab) bound to cleaved or uncleaved H7 HA trimers. Together, these data reveal a previously unappreciated antigenic determinant on HA that, while somewhat inaccessible, may be exploited as a new target for vaccine design, given its relative conservation and structural importance. These studies also indicate that the HA trimer is likely sampling subtly different prefusion conformations that may provide clues about the early aspects of the conformational changes that accompany the fusion process.

## Results

**Fig 1:**
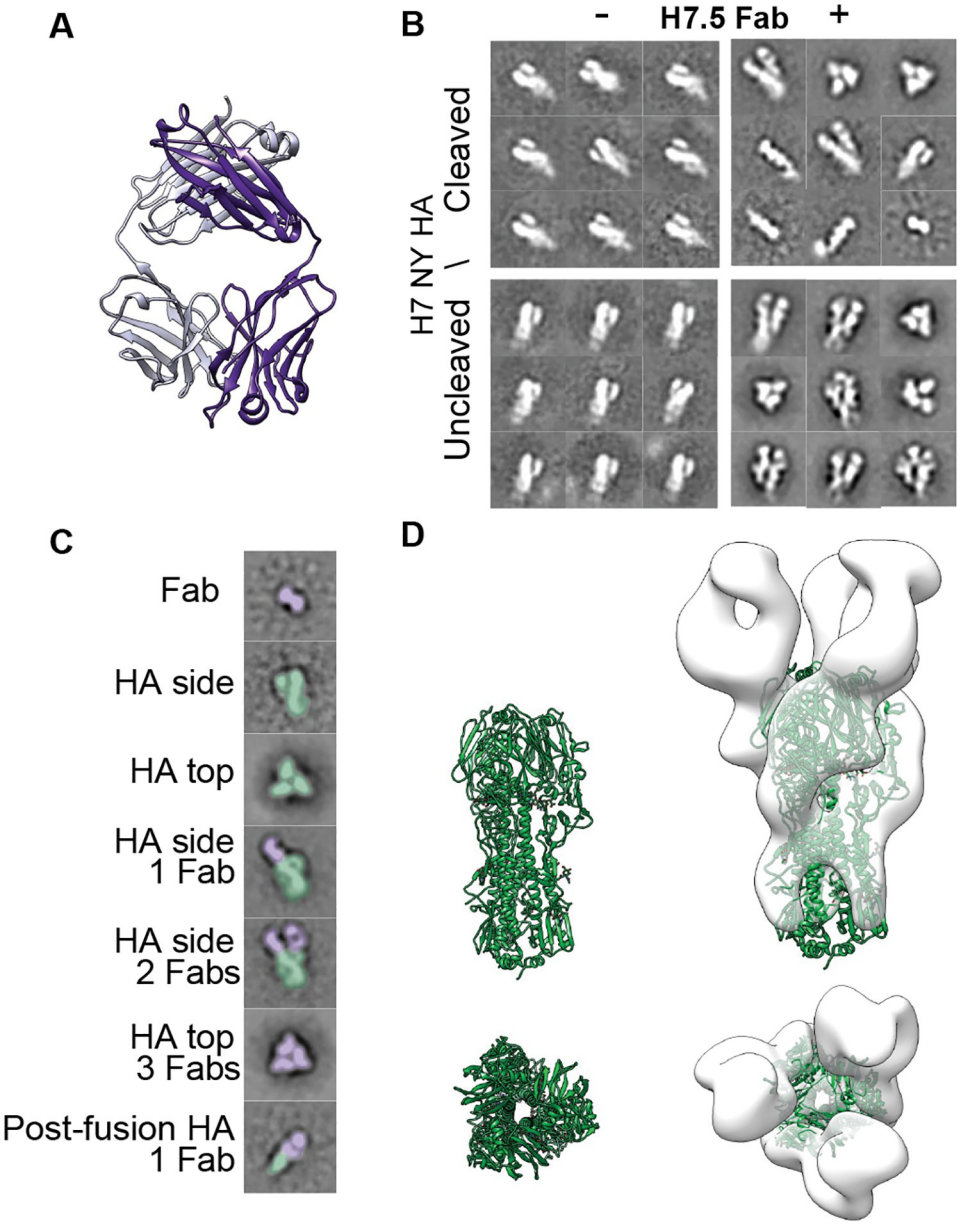
H7.5 Fab crystal structure and nsEM 2D class averages of H7.5 complexed with H7 HA. (A) H7.5 Fab-heavy (purple) and light-chain (light purple) crystal structure at 2.0 Å resolution. (B) nsEM 2D classes of cleaved and uncleaved H7 NY with and without H7.5 Fab. (C) Example 2D class averages showing range of observations seen after adding H7.5 Fab to H7 HA trimer. The antibody is colored purple and H7 HA colored green. (D) Side and top views of H7 HA1/H2 trimer (green, PDB 3M5G) (left panel), docked into a low-resolution negative-stain 3D reconstruction (white surface) of uncleaved H7 NY HA with H7.5 Fab (right panel). Based on the docking, the H7.5-bound HA0 trimer appears to be in a somewhat different conformation than the unliganded HA crystal structure. EM, electron microscopy; Fab, fragment antigen binding; HA, hemagglutinin; nsEM, negative-stain EM; PDB, Protein Data Bank.

Antibody H7.5 was described in our previous study that identified several broadly neutralizing antibodies isolated from the peripheral blood B cells of donors who participated in a vaccination trial with monovalent, inactivated influenza (H7N9), A/Shanghai/02/2013 vaccine candidate (DMID13-0033) [10]. mAb H7.5 was shown to neutralize H7 viruses and exhibit strong inhibition of hemagglutination activity against a variety of H7 HAs. Preliminary analysis revealed that H7.5 recognizes multiple strains of H7 HAs with K_d_ (dissociation constant) of less than 0.1 nM, even in the monovalent Fab form, when measured using biolayer interferometry (S1 Fig). The breadth and high affinity for the emerging H7N9 viruses made H7.5 an interesting target for in-depth structural studies. Hence, we first determined the structure of unliganded H7.5 Fab by X-ray crystallography at 2.0 Å resolution (Fig 1A, Table 1), but its complex with H7 HA could not be obtained, despite extensive screening of crystallization conditions.

**Table 1:** X-ray data collection and refinement statistics for H7.5 Fab.

Data collection	H7.5 Fab
Beamline	APS 23 ID-D
Wavelength (Å)	1.03320
Space group	P22_1_2_1_
Unit cell parameters (Å, °)	*a* = 65.5 *b* = 99.0, *c* = 208.6; α = β = γ = 90
Resolution (Å)	50.00–2.00 (2.03–2.00)
Observations	624,618
Unique reflections	91,327 (4,421)
Redundancy	6.8 (6.3)
Completeness (%)	98.5 (96.6)
<*I/σ*_*I*_>	21.0 (1.5)
*R*_sym_ ^a^	0.11 (0.74)
*R*_pim_^b^ CC_1/2_^c^	0.05 (0.32) 1.00 (0.80)
**Refinement statistics**	
Resolution (Å)	49.52–2.00 (2.02–2.00)
Refs used in refinement	91,238 (2,795)
*R*_work_ (%)^d^	19.9
*R*_free_ (%)^e^	20.2
Protein atoms	6,555
Waters	746
Other	0
***B* value (Å**^**2**^**)**	
Average *B* value Protein Water	36 35 43
Wilson *B*	31
**RMSD**	
Bond length (Å)	0.009
Bond angles (°)	1.144
**Ramachandran plots (%)**^f^	
Favored	97.5
Outliers	0.2
PDB	6BTJ

Abbreviations: APS, Advanced Photon Source; Fab, fragment antigen binding; PDB, Protein Data Bank; RMSD, root-mean-square deviation.

Values in parentheses are for the highest-resolution shell.

^a^*R*_sym_ = Σ_*hkl*_ Σ_*i*_ | *I*_*hkl*,*i*_ − <*I*_*hkl*_> | / Σ_*hkl*_ Σ_*i*_ *I*_*hkl*,*I*_ and

^b^*R*_pim_ = Σ_*hkl*_ (1/(*n* − 1))^1/2^ Σ_*i*_ | *I*_*hkl*,*i*_ − <*I*_*hkl*_> | /Σ_*hkl*_ Σ_*i*_ *I*_*hkl*,*i*_, where *I*_*hkl*,*i*_ is the scaled intensity of the *i*^th^ measurement of reflection h, k, l, < *I*_*hkl*_ > is the average intensity for that reflection, and *n* is the redundancy (1).

^c^CC_1/2_ = Pearson Correlation Coefficient between two random half data sets.

^d^*R*_work_ = Σ_*hkl*_ | *F*_o_ − *F*_c_ | / Σ_*hkl*_ | *F*_o_ | × 100.

^e^*R*_free_ was calculated as for *R*_work_ but on a test set comprising 5% of the data excluded from refinement.

^f^Calculated using MolProbity (2).

In parallel to the crystallization efforts, we used nsEM to characterize H7.5 Fab in complex with HA protein from different H7 strains. The difficulty in obtaining crystals of the complex became immediately apparent upon inspection of the complexes in nsEM (Fig 1B and 1C). Reference-free 2D classification of cleaved H7 NY HA (A/New York/107/2003) in complex with H7.5 Fab showed substantial heterogeneity relative to previously observed prefusion complexes of HA bound to other head-binding antibodies [10] (Fig 1B and 1C). In nsEM 2D classes, we observed several different phenomena, including variable stoichiometries of bound Fabs and heterogeneous species that were difficult to interpret (Fig 1B and 1C). The effect of H7.5 was observed consistently with both cleaved and uncleaved forms of H7 HA from multiple strains including H7 NY HA (A/New York/107/2003), H7 Sh2 HA (A/Shanghai/2/2013), and H7 NL HA (A/Netherlands/219/2003) (S2 Fig). We therefore attempted to capture intermediate conformations of the complex by incubating the cleaved trimer with H7.5 for shorter periods of time. Indeed, a 5-minute incubation resulted in enough reliable observations to deduce that H7.5 bound to the HA1 head but induced an unfamiliar structural phenotype in our 2D classes (Fig 1B). Within a single data set, all combinations of the H7.5/H7 HA complex stoichiometry were observed (from zero to three Fabs per trimer and individual protomers bound to H7.5 Fab) that probed the conformational landscape of HA (Fig 1B and 1C). Relative to other prefusion HA structures, the HA1 heads appeared separated, with H7.5 apparently prying them apart or stabilizing a more open form of the HA trimer (Fig 1D).

Unlike cleaved H7 HA trimers that rapidly fell apart into protomers, uncleaved H7 HA (HA0) remained in a trimeric conformation even after overnight incubation with H7.5 (Fig 1B), although the separation of the HA heads make it distinct from the closed, prefusion conformation observed in a large number of crystal structures. This relatively stable complex enabled us to image a larger number of intact particles. In comparisons of 2D classes of cleaved and uncleaved trimeric complexes of H7 HA with H7.5, there appears to be no large difference whether zero, one, two, or three Fabs are bound (Fig 1B). Hence, the uncleaved H7 HA, which is stable in the presence of H7.5, is likely to adopt an overall similar structure to the cleaved H7 HA prior to H7.5-induced degradation. We do note that our higher resolution analysis discussed below does indicate that there are likely subtle differences in the molecular interactions between protomers of cleaved and uncleaved HA, depending on the stoichiometry of H7.5 binding.

Our EM data demonstrated that H7.5 had a substantial disruptive effect on the prefusion structure of the HA trimer. To further validate the influence of H7.5 on the HA trimer structure, we investigated the susceptibility of H7 HA to trypsin protease digestion with or without H7.5 bound. Indeed, this experiment showed that addition of H7.5 Fab to H7 Sh2 HA in the presence of trypsin resulted in degradation of HA into many peptidic fragments (S3 Fig). When H7.5 Fab was added to H7 Sh2 HA, 0.1% trypsin was enough to induce the cleavage of H7 Sh2 HA, and 2% was sufficient to completely degrade the H7 HA trimer. Without H7.5 Fab, the H7 Sh2 HA trimers remained resistant to protease cleavage up to a trypsin concentration of 2%. These data indicate that H7.5 may prematurely trigger structural changes or fluctuations of H7 HA trimers that acquire protease-sensitive conformations, which is reminiscent of the typical behavior of HA trimers in low-pH environments [11,12].

**Fig 2:**
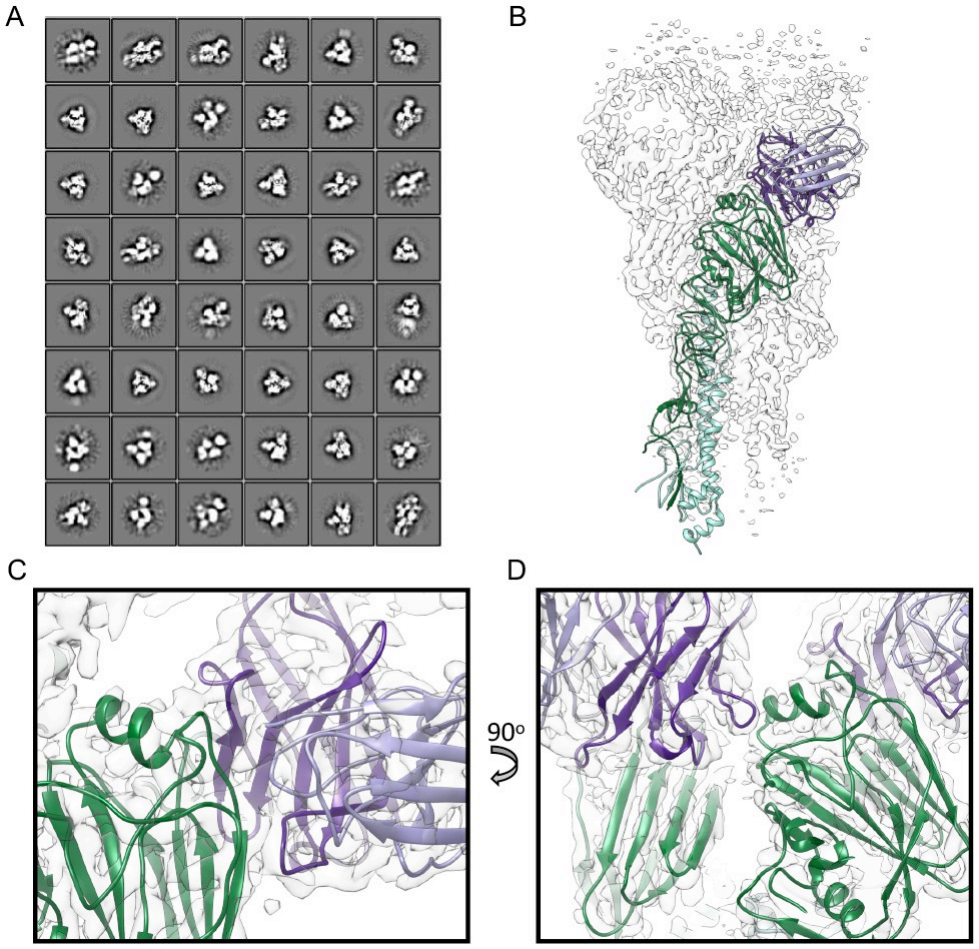
2D and 3D cryoEM image analysis and reconstruction of Fab H7.5 complexed with uncleaved H7 NY HA. (A) 2D class averages produced in Relion of uncleaved H7 NY HA with H7.5 Fab. Secondary structure features are visible in a variety of different views of the complex. (B) 3D reconstruction (transparent white surface) and H7.5/H7 NY HA atomic model (H7 HA1 in green, HA2 in cyan, and H7.5 in light and dark purple). (C) Close-up view of H7.5 variable region and HA1 head. (D) Close-up view of cross-protomer interaction of H7.5 with neighboring receptor-binding domain. cryoEM, cryo-electron microscopy; Fab, fragment antigen binding; HA, hemagglutinin.

To interrogate the structure of H7.5 bound to H7 HA at a higher resolution, we employed single particle cryoEM of the uncleaved H7 NY HA in complex with H7.5 Fab. Reference-free 2D classification resulted in many different views of the complex, and secondary structure features were clearly visible (Fig 2A). The cryoEM 2D classes of the complex in vitreous ice were similar to those observed in nsEM, although the majority of the classes appeared to have three Fabs bound to the HA trimers. Projection images corresponding to meaningful 2D classes were subjected to iterative angular reconstitution and reconstruction. The resulting density map exhibited significant heterogeneity in the Fab densities attached to the head as well as in the membrane-proximal part of the stem. 3D classification was then performed, and among the resulting classes, one class was characterized by having well-resolved Fabs bound to the head domain. Data in this class were subjected to further refinement resulting in a 3-fold symmetric map with a global resolution of approximately 3.5 Å (EMDB-9139, PDB 6MLM) that is well resolved in all but the membrane-proximal stem region (Fig 2B, Table 2). An initial homology model was created using an X-ray structure of the H7 HA1/HA2 protomer (A/New York/30732-1/2005, 3M5G) [13], combined with our X-ray structure of the H7.5 Fab, and then individually docked into our cryoEM density map exhibiting a nice fit (Fig 2B, 2C and 2D). Next, iterative rebuilding and refinement was carried out in Rosetta [14], yielding a high-resolution atomic model. Since the membrane-proximal part of the map was not well resolved, a protocol for density-subtracted refinement of this region alone was pursued, resulting in a local density map at approximately 3.9 Å resolution (S4 Fig). The membrane-proximal part of the model was refined under constraints of this density map, and an atomic model was created from combining the two builds. To confirm that local (Brownian) motion was responsible for the disorder in the membrane-proximal region—as opposed to the two maps displaying H7 HA in different conformations—coordinates assigned in the density-subtracted local refinement of cryoEM data were applied to the original data, and a reconstruction was performed. Indeed, density corresponding to HA1 and to the parts of HA2 not included in local refinement were clearly visible, albeit at an expected lower resolution (S5 Fig), thereby justifying combining the two builds into a meaningful global conformation supported by our experimental observation.

**Table 2:** EM data collection and refinement statistics.

Map	H7 HA0	H7 HA0 Stem Base	H7 NL HA1/2-3 H7.5 Fabs	H7 NL HA1/2-2 H7.5 Fabs	H7 SH HA1/2-3 H7.5 Fabs	H7 NY 1/2-3 H7.5
**EMDB ID**	EMD-9139	EMD-9139	EMD-9143	EMD-9145	EMD-9142	EMD-9144
**Data collection**						
Microscope	FEI Titan Krios	FEI Titan Krios	FEI Talos Arctica	FEI Talos Arctica	FEI Talos Arctica	FEI Technai Spirit
Voltage (kV)	300	300	200	200	200	120
Detector	Gatan K2 Summit	Gatan K2 Summit	Gatan K2 Summit	Gatan K2 Summit	Gatan K2 Summit	TemCam F416
Recording mode	Counting	Counting	Counting	Counting	Counting	Single exposure
Magnification (incl. postmagnification)	49,020	49,020	43,478	43,478	43,478	76,098
Movie micrograph pixelsize (Å)	1.02	1.02	1.15	1.15	1.15	2.05
Dose rate (e^−^/[(camera pixel)*s])	10	10	8.375	8.375	7.576	25
Number of frames per movie micrograph	35	35	32	32	32	1
Frame exposure time (ms)	200	200	250	250	250	500
Movie micrograph exposure time (s)	7	7	9	9	8	−0.5
Total dose (e^−^/Å^2^)	67	67	65	65	60	25
Defocus range (μm)	0.4–4.0	0.4–4.0	0.4–5.0	0.4–5.0	0.5–4.0	−1.5
**EM data processing**						
Number of movie micrographs	1,324	1,324				
Number of molecular projection images in map	30,032	30,032	25,146	9,682	32,117	
Symmetry	C3	C3	C1	C1	C1	C3
Map resolution (FSC 0.143; Å)	3.5	3.9	9.2	10.2	7.4	20
Map sharpening *B* factor (Å^2^)	−100	−157	−259	−109	−296	
**Structure building and validation**						
**PDB ID**	6MLM					
Number of atoms in deposited model	16,710	5,040				
HA1	2,399	517				
HA2	1,370	1,163				
Glycans	42	0				
MolProbity score	1.23 (99%)	1.66 (91%)				
Clashscore	2.41	4.96				
EMRinger score	2.71	1.14				
Deviations from ideal, RMSD						
Bond length (Å)	0.013	0.015				
Bond angles (°)	1.17	1.4				
Ramachandran plot						
Favored (%)	96.7	94.0				
Allowed (%)	3.0	5.0				
Outliers (%)	0.3	1.0				

Abbreviations: EM, electron microscopy; EMDB, Electron Microscopy Data Bank; FSC, Fourier shell correlation; HA, hemagglutinin; PDB, Protein Data Bank; RMSD, root-mean-square deviation.

**Fig 3:**
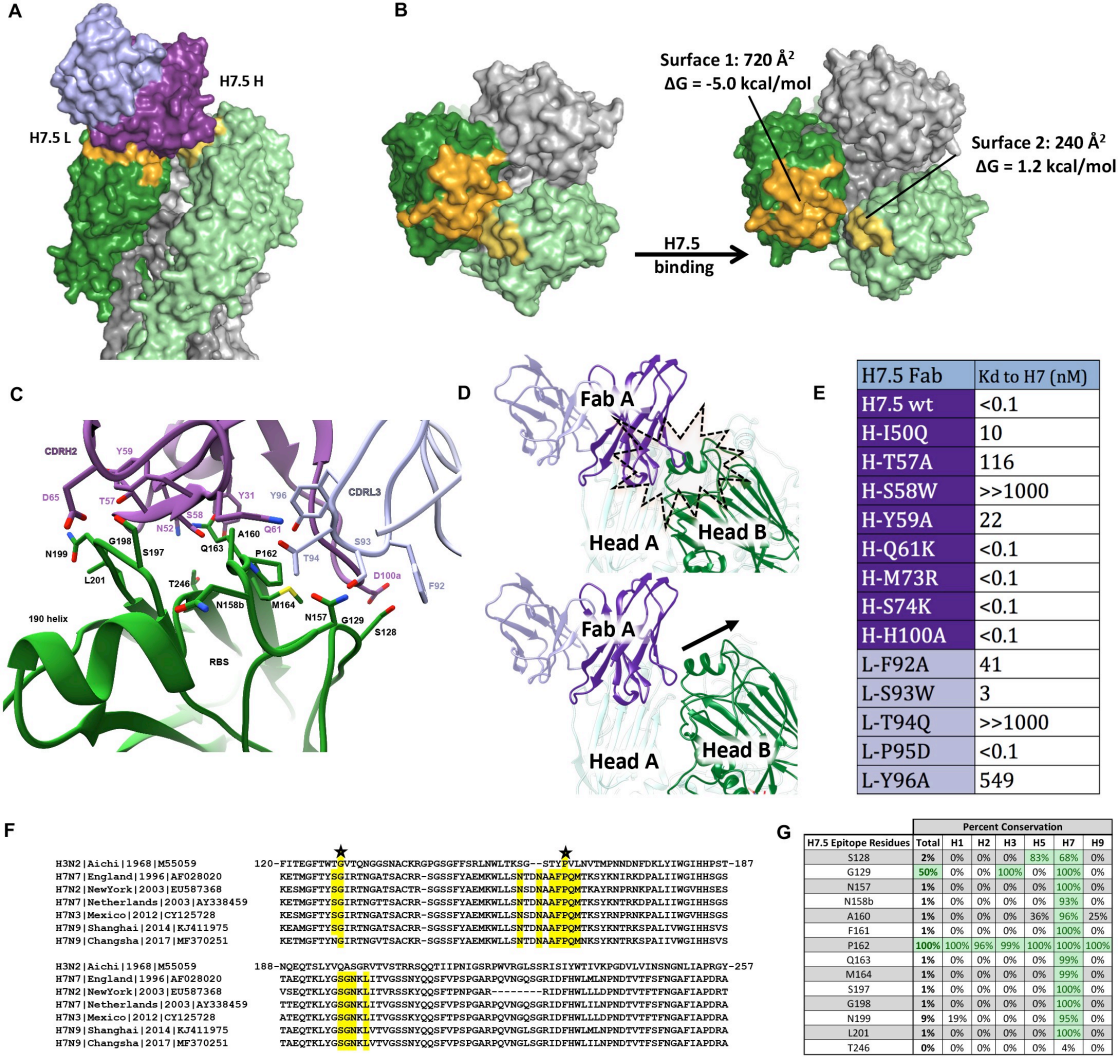
HA-protomer movement and mutagenesis. Each H7.5 Fab was found to recognize a primary epitope on one HA1 subunit and a second smaller region on an adjacent HA1 subunit. (A) The variable domain of one H7.5 Fab is shown as a purple surface for the heavy chain and as a light purple surface for the light chain. The HA1 subunit primarily recognized by H7.5 Fab is shown as a dark green surface, with the epitope surface color in orange (surface 1). The adjacent subunit that is also contacted by the same Fab was colored in light green, with the epitope region in yellow (surface 2). (B) Top view of the H7.5 epitope on H7 HA before and after H7.5 binding. The estimated buried area surfaces on each HA1 subunit and its predicted Gibbs free energies upon H7.5 binding were analyzed. (C) Interaction between Fab H7.5 and HA primary binding surface 1. The H7.5 antibody is shown as main-chain trace, with the interacting residues that are critical to the H7 recognition shown in sticks. The HA1 subunit with surface 1 is shown in a main-chain trace. The main chain and side chain of H7 HA residues that form direct contacts with H7.5 are shown in stick representation. (D) Interaction between two adjacent protomers and a single Fab. When the H7.5 antibody (purple) is docked onto the closed HA (green) head conformation exhibited in 3M5G, there is a clash with the adjacent protomer (top panel). When the 3M5G HA head is morphed into our model, the increased spacing between the heads alleviates this clash, thereby accommodating the CDRH3 loop (bottom panel). (E) The K_d_ values of H7.5 or mutated H7.5 Fab binding to the HA1 subunit of H7 (A/Shanghai/2/2013) by biolayer interferometry. (F) Sequence alignment of H7 strains with H3N2 reference strain showing the H7.5 epitope (highlighted in yellow) is well conserved among H7. (G) Amino acid sequence analysis of H7.5 epitope among 13,880 influenza A HA sequences obtained from www.fludb.org showing poor conservation in the H7.5 epitope in subtypes other than H7. CDRH3, complementarity determining region heavy chain 3; Fab, fragment antigen binding; HA, hemagglutinin.

The epitope on the H7 HA trimer recognized by H7.5 Fab was delineated based on the cryoEM model (Fig 3A). Intriguingly, the H7.5 antibody was found to simultaneously interact with two separated surfaces on two adjacent HA protomers (Figs 2D and 3B). Moreover, our model illustrated that the epitope recognized by H7.5 is not completely accessible when all three HA1 heads are close together, as observed in the apo cleaved H7 trimer crystal structure. Interestingly, the heavy-chain framework region 3 (H-FR3) of H7.5 was juxtaposed to the RBS of an adjacent HA protomer (S6 Fig), and in a closed-trimer model, CDRH3 of H7.5 would clash with residues 189 to 194 in the 190 helix of the adjacent protomer (Fig 3D).

Compared to the apo cleaved H7 trimer model, the buried interprotomer surfaces in the H7.5 epitope were separated substantially from each other in the H7.5-bound H7 NY HA model (Fig 3B). The recessed nature of this epitope suggests that one of the surfaces was likely the primary binding interface recognized by H7.5 antibodies, while the other surface allosterically alters upon antibody binding. Indeed, further interface analysis revealed that the HA surface interacting with CDRH2 and CDRL3 of H7.5 (surface 1) generates a total buried surface area of 720 Å^2^, with substantial favorable energy estimated to be −5 kcal/mol and an extensive hydrogen bond network between H7.5 and the HA in surface 1 (Fig 3B and 3C). In contrast, the surface between the adjacent HA protomer and H-FR3 of H7.5 (surface 2) is significantly smaller (only 240 Å^2^) and appears to be energetically unfavorable with a ΔG (change in Gibbs free energy) of +1.2 kcal/mol (Fig 3B and S6 Fig).

To confirm the structural observations, we performed mutagenesis studies of residues in the antibody paratope by individually reverting the residues to the corresponding germline residues and measuring the affinity changes of the H7.5 mutants to H7 Sh2 HA. Consistent with the interface analysis, mutations in CDRH2 and CDRL3, particularly H-S58W, H-T57A, L-T94Q, and L-Y96A of H7.5, drastically reduced binding to H7, while mutations in H-FR3 of H7.5, namely H-M73R and H-S74K, which are proximal to surface 2, did not impact the binding affinity (Fig 3E and S6 Fig). These results strongly support the hypothesis that surface 1 is the primary binding surface driven by favorable energy changes, while surface 2 is a collateral result of the antibody-binding event.

Although mutations in H-FR3 or CDRH3 of the H7.5 antibody did not affect binding of the antibody itself, Fab binding requires the adjacent protomer to be displaced from the tight 3-fold axis at the apex of the trimer to reveal the full epitope (Fig 3D and S6 Fig). The H7.5 epitope therefore includes residues in the inter-HA head contact region (Fig 3E and S6 Fig). Notably, these residues are conserved across all human and zoonotic H7 strains from 1996–2017 (Fig 3F and 3G and S7 Fig) but not amongst other strains, with the exception of proline at position 62. (S7 Fig). Binding of all three Fabs ultimately results in a conformation of HA with the heads splayed apart.

**Fig 4:**
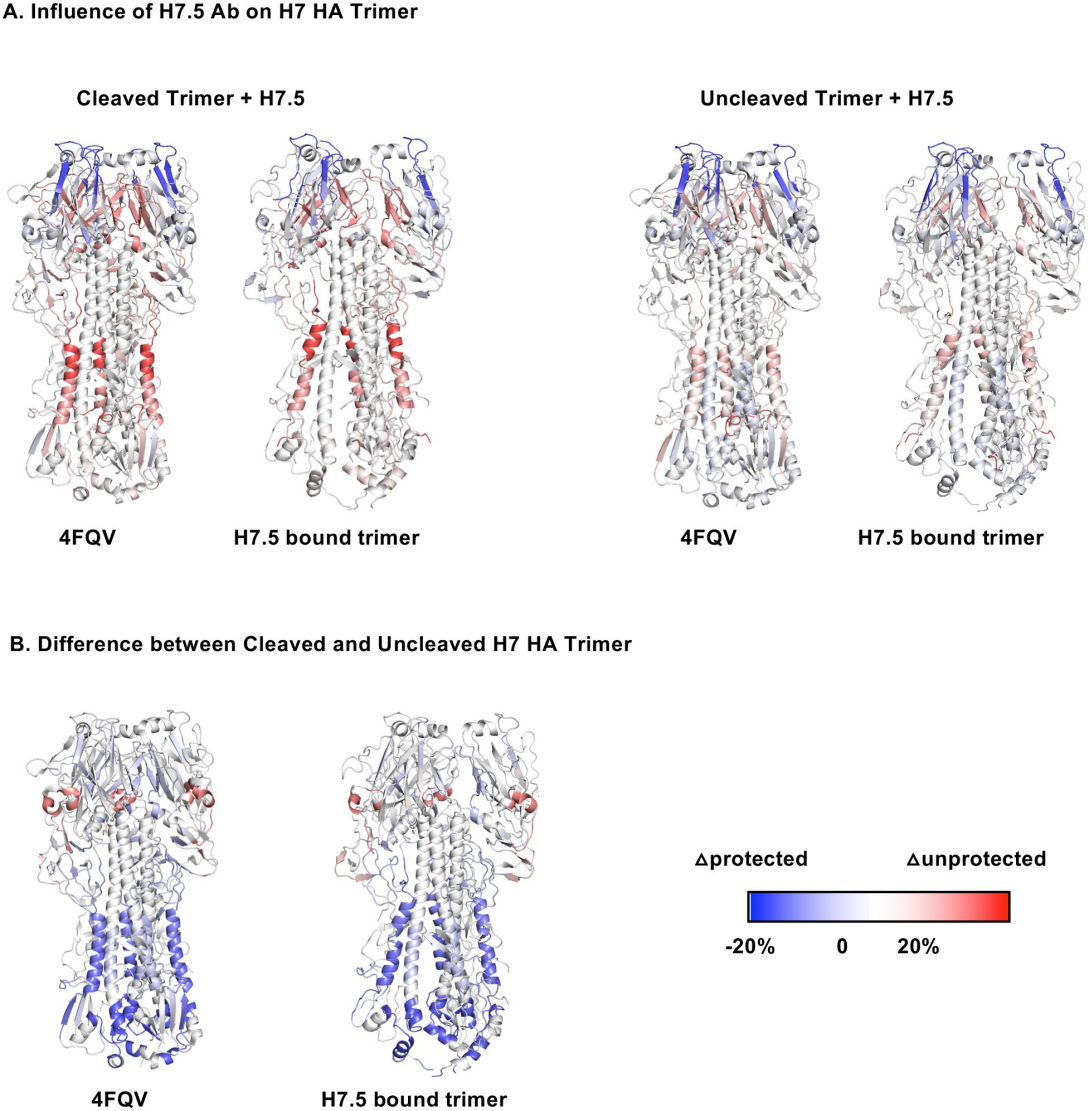
HDX-MS analysis of H7 HA trimer–H7.5 complexes. (A) Difference in HDX profiles of cleaved (left) and uncleaved (right) H7 HA upon H7.5 binding, mapped onto closed (4FQV) and H7.5-bound (H7.5/H7 NY HA) model of H7 HA. The epitope of H7.5 in the HA head shows greater protection, consistent with antibody binding. H7.5 binding also induces greater exchange in the HA head region interprotomer interface as well as the stem, consistent with structural changes in our models. The effects are stronger in the cleaved HA, consistent with H7.5 induction of trimer dissociation. (B) Regions that are more (blue) or less (red) protected in cleaved H7 HA relative to uncleaved H7 HA are mapped onto closed (4FQV) and H7.5-bound (H7.5/H7 NY HA) model of H7 HA. The stem region of the cleaved HA is more protected from exchange than the uncleaved stem, consistent with maturation of the structure and burying of the fusion peptide in the core of the trimer upon cleavage. HA, hemagglutinin; HDX-MS, hydrogen–deuterium exchange mass spectrometry.

To further probe the conformational dynamics of the H7 HA when it interacts with H7.5 Fab, we performed hydrogen–deuterium exchange mass spectrometry (HDX-MS) studies on cleaved or uncleaved H7 NL HA in the presence or absence of H7.5 Fab. The differential HDX-MS results of H7 NL HA and H7 NL HA/H7.5 complex were mapped onto the H7 NL HA crystal structure (PDB 4FQV) (Fig 4A). The HDX-MS data revealed two protected regions (colored in blue) in HA1 of H7 HA upon H7.5 binding. These two discontinuous regions (residues E150–K166 and residues S197–L201) form the potential binding epitope, which is consistent with the cryoEM results (Fig 3C). Moreover, HDX-MS experiments performed on cleaved H7 NL HA alone indicated a low level of exchange in one of the regions (residues E150–K166), suggesting less solvent accessibility in the binding epitope region. On the other hand, H7.5 binding led to deprotection of regions (colored in red) in HA1 and HA2, which indicated that HA became more conformationally flexible to accommodate the binding of multiple H7.5 Fabs.

The structural dynamic differences between cleaved and uncleaved H7 NL HA were also compared (Fig 4B). The HDX-MS results indicated that the cleaved H7 NL HA showed more protection (colored in blue) in the HA2 subunit compared to uncleaved H7 NL HA, which is more prone to protease cleavage. This finding is consistent with the helical domains in cleaved HA being more stable and is likely related to the maturation of HA from uncleaved HA0 to HA1 and HA2. Detailed results are shown in S8 Fig.

**Fig 5:**
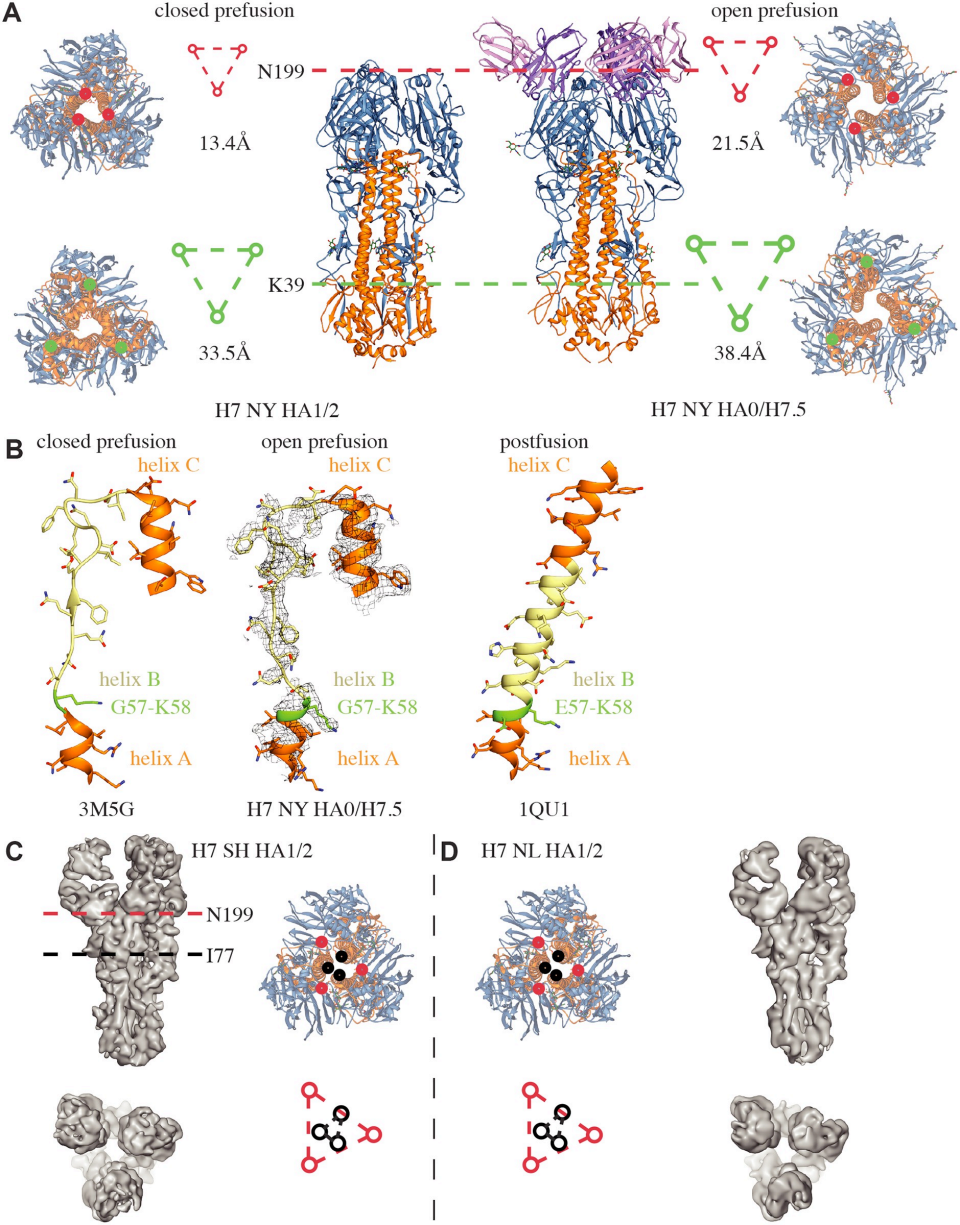
Comparison of H7 strains and movement between unliganded and H7.5 liganded HA trimers. (A) Cross sections of open prefusion trimer (H7.5/H7 NY HA) shows separation in the head domain at N199 (red triangle) and in the stem at K39 (green triangle) when compared to closed prefusion trimer (H7 NY HA) (left side). (B) Residues G57–K58 in HA2 show a transition into a helical conformation after H7.5 binds, as seen previously in the postfusion conformation (PDB 1QU1). Moderate-resolution cryoEM reconstructions of cleaved H7 Sh2 HA1/2 (C) and H7 NL HA1/2 (D). Individual HA1/2 protomers from HA (PDB 3M5G) were fit into the reconstructions to generate pseudoatomic models. The red and black triangles indicate the spacing of residues N199 (red) and I77 (black) based on the models. Similar to the uncleaved structure in complex with H7.5 shown in (A), the heads move apart relative to prefusion crystal structure. The density maps, reconstructed asymmetrically, appear to have a subtle similar asymmetry, although the limited resolution prevents detailed analysis. cryoEM, cryo-electron microscopy; HA, hemagglutinin; PDB, Protein Data Bank.

To trap additional structural intermediates of HA, we next attempted cryoEM reconstructions of H7.5 Fab bound to cleaved H7 Sh2 HA and H7 NL HA, which differ greatly in season and global location of isolation. Guided by our nsEM experiments (Fig 1B), we succeeded in capturing an intermediate structure of cleaved H7 HA using a short incubation time with H7.5 Fab immediately prior to sample vitrification for EM experiments. The predominant classes that we observed had three H7.5 Fabs bound to H7 HA and were able to generate asymmetric 3D reconstructions at approximately 7.4 and approximately 9.2 Å resolution (EMDB-9142, EMDB-9143) (Fig 5C and 5D, S9 Fig and Table 2). Similar to the high-resolution structure of H7.5 bound to uncleaved H7 HA, we still observed movement of the HA1 heads away from the apical 3-fold axis in the cleaved H7 HAs. Further, in all reconstructions of the cleaved H7 HAs, one of the HA1 heads appeared to be splayed out further than the other two (Fig 5C and 5D). In the case of cleaved H7 NL HA, we also refined a class with only two Fabs bound (S9C Fig).

**Fig 6:**
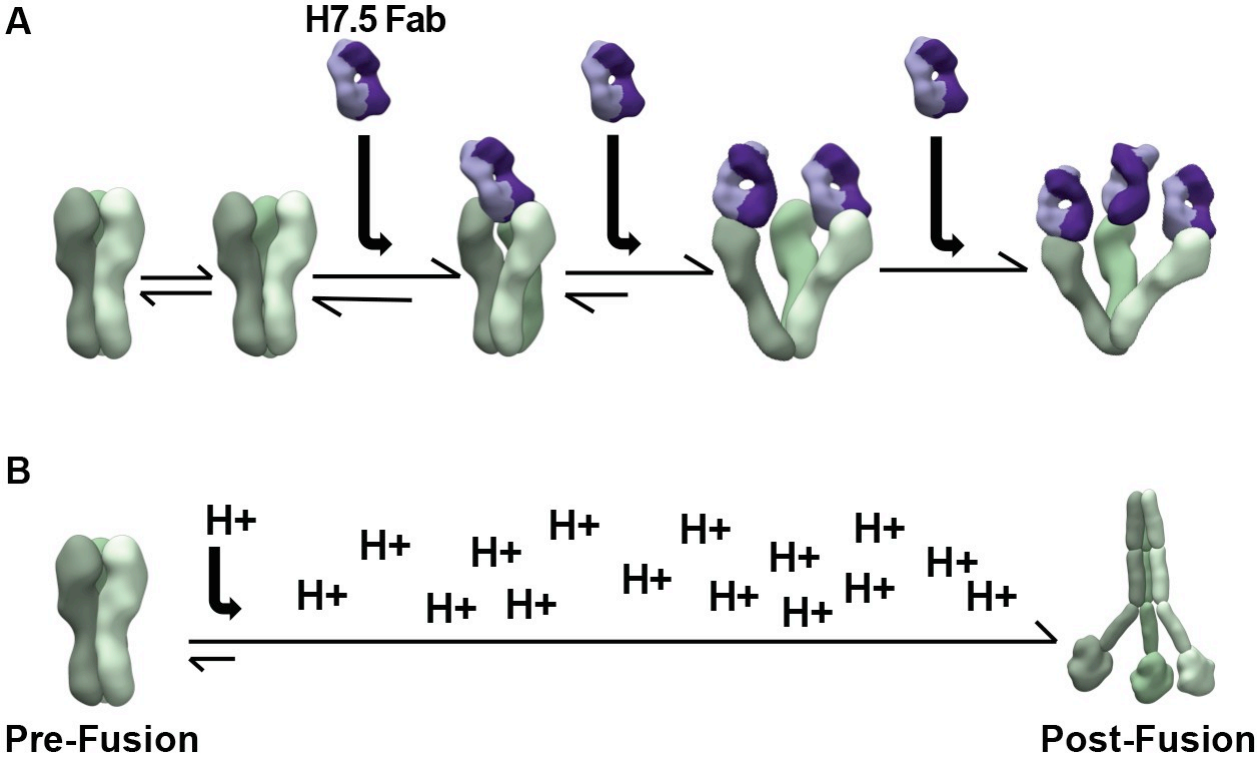
Movement of protomers as antibodies bind and induce the trimer to fall apart. (A) HA trimer heads breathe in equilibrium. When an H7.5 antibody is present (purple), it binds to its epitope and prevents reclosing of the HA head, allowing more antibodies to bind. (B) During the fusion process in the endosome, the low pH environment triggers conformational rearrangements in HA. HA, hemagglutinin.

We next compared our high-resolution cryoEM-derived model of the H7.5 bound to H7 NY HA, with separated heads to a crystal structure of HA in the canonical closed prefusion conformation (PDB 3M5G). Within a single protomer, our hybrid docking revealed that the HA1 movement was coupled to a subtle change in its position relative to HA2 (Fig 5). At the trimer level, head separation was accompanied by movement in the HA2 central helices (Fig 5A). Hence, the HA head motions appear to be communicated downward to the stem, resulting in a small separation of the stem protomers. We hypothesize that this looser packing may trigger events that ultimately liberate the fusion peptide or otherwise destabilize the HA trimer and would lead to initiation of the downhill fusion process, a phenotype that would explain our initial nsEM results with the cleaved HA showing dissociation of the trimer into protomers after incubation with H7.5. Additionally, the head separation correlates with HA2 residues G57–K58 adopting an α-helical conformation (Fig 5B). These two amino acids were observed previously as a random coil following the C-terminus of HA2 helix A [13]. The segment (residues G57–I73) connects helix A with the HA2 central helix; in the stable, trimeric HA2 postfusion state, this segment was observed to be α-helical, forming a continuous extended helix with helix A and the central helix [15]. The α-helical organization of residues G57–K58 also correlates with further separation of the membrane-proximal H7 stem. Thus, this structure likely represents an intermediate conformational state of the class I fusion protein of influenza virus. Fig 6 summarizes the results and the events leading up to the trimer dissociation when H7.5 is added.

## Discussion

With the ever-present risk of new avian influenza outbreaks looming, it is important to explore novel ways to target influenza. The recently identified H7.5 recognizes a new conserved epitope on the HA head that can be targeted by neutralizing antibodies. The H7.5 epitope, conserved throughout H7 HA, resides at the HA head protomer–protomer interface and is only transiently accessible. Neutralizing antibodies that bind the HA1 head often target the RBS, blocking HA from interacting with sialic acid on host cells [16]. Although H7.5 does not bind directly to the RBS, it does however block sialic acid binding to HA [10] and potently blocks intact HA from approaching the cell surface.

The prefusion structure of HA has been described extensively, with HA1 and HA2 tightly interacting in a compact arrangement in both cleaved and uncleaved structures [10,17,18]. Portions of the postfusion HA structure have also been crystallized and described [19,20]. Interestingly, mAb H7.5 induces premature dissociation without exposure to low pH, and the somewhat open conformation of the receptor-binding domains may be reminiscent of early steps in the influenza virus HA-mediated membrane-fusion process. After H7.5 binds, it likely induces progressive opening of the trimer, and as more antibodies bind, the trimer is pushed over the activation barrier required for transition into a postfusion conformation [1]. In fact, there are other recently reported HA head–directed antibodies that have indirectly been shown to induce a similar phenotype [21,22]. Notably, these antibodies do not bind to epitopes that overlap with H7.5 (S10 Fig). Antibody-induced decay of HA, via different head-binding antibodies, likely represents a new mechanism of influenza neutralization inhibition.

To date, HA has been shown to adopt a stable, closed prefusion conformation [17,23]. However, despite significant effort, intermediates in the fusion process have eluded high-resolution characterization. It is likely that these intermediates are highly unstable and rapidly transition to the postfusion form [24]. Altogether, our cryoEM studies reveal that HA appears to be somewhat dynamic in its prefusion state, wherein protomers are able to undergo structural fluctuations. Indeed, we observed movement throughout the HA molecule, including fluctuations in the HA2 stem. The inferred movements of the HA heads to enable antibody H7.5 binding suggest that the HA1 head is “breathing,” and this motion is reminiscent of conformational masking described for the HIV envelope glycoprotein [25–27]. Indeed, a very recent study using single-molecule FRET (Förster resonance energy transfer) observed that H5 HA undergoes reversible conformational changes [28]. The structural fluctuations observed in our study, as well as the smFRET study, indicate that movement of the HA1 heads alters interactions between the HA1/HA2 stem portion and the fusion peptide, which reside in the trimer core. HDX-MS studies of H3 HA liberated from whole virions demonstrated a stepwise progression of acid-induced fusion peptide liberation followed by HA head separation [29]. Whether these movements are similarly recapitulated amongst all strains of HA remains to be investigated, but there are likely to be conserved and coordinated motions, given the critical role that the HA-fusion machine plays in viral entry. Thus, there may be ways to exploit these structural fluctuations and access conserved epitopes that have not previously been appreciated relative to the more canonical RBS and stem antibodies.

## Materials and methods

### Preparation of recombinant H7.5 antibodies

H7.5 Fab for crystallization was prepared as previously described [30]. In brief, the heavy and light chains of H7.5 were cloned independently into the phCMV3 vector and fused with an N-terminal IgK secretion signal. A His_6_ tag was added to the C-terminus of the Fab heavy chain. Recombinant cDNAs encoding the Fab heavy and light chains were purified and cotransfected into 293F cells by 293fectin (Invitrogen). After 6–7 days of expression at 37 °C, the Fabs were purified from the supernatant by Ni-NTA Superflow (Qiagen) and monoS chromatography (GE Healthcare).

### Crystallization and structure determination of H7.5 Fab

H7.5 Fab was concentrated to 10.0 mg/mL in the buffer of 50 mM NaOAc (pH 5.5) for crystallization screening on our high-throughput robotic CrystalMation system (Rigaku) at The Scripps Research Institute (TSRI) using sitting-drop vapor diffusion. The best crystals grew in the well with 0.1 M MES (pH 6.5, 0.01), M cobalt chloride, and 1.8 M ammonium sulfate as mother liquor at 4 °C. Crystals were cryoprotected with mother liquor supplemented with 15% ethylene glycol. X-ray diffraction data were collected to 2.00 Å resolution on beamline 23-ID-D at the Advanced Photon Source (APS). The diffraction data were processed with HKL2000 [31], and the structure was determined by molecular replacement with initial models of PDB 3N9G and PDB 4KMT in Phaser [32]. Refinements were carried out in PHENIX [33], and model rebuilding was performed manually in Coot [34] and the model validated by MolProbity [35].

### Structural analysis

Hydrogen bond and buried molecular surface analyses were calculated using the PDBePISA server of EMBL-EBL. Structure figures were generated by MacPyMOL (DeLano Scientific, LLC) and UCSF Chimera package. Chimera is developed by the Resource for Biocomputing, Visualization, and Informatics at the University of California, San Francisco (supported by NIGMS P41-GM103311) [36].

### Preparation of H7 HA

Baculovirus-expressed HA was prepared for the study as previously described [3,37,38]. In brief, the HA ectodomain sequence was cloned into the pFastBac vector with an N-terminal gp67 secretion signal peptide, a C-terminal BirA biotinylation site, thrombin cleavage site, foldon trimerization domain, and His_6_ tag. Recombinant bacmid DNA was generated via the Bac-to-Bac system (Invitrogen), and Baculovirus was generated by transfecting purified bacmid DNA into Sf9 cells. HAs were expressed by infecting the High Five cells with the recombinant virus, shaking at 110 rpm for 72 hours at 28 °C. The secreted HAs were purified from the supernatant via Ni-NTA Superflow (Qiagen) and gel filtration (to collect only the HA trimer). The HA trimer was concentrated in the buffer of 20 mM Tris-HCl (pH 8.0) and 150 mM NaCl and aliquoted for storage.

### Trypsin digestion for protease susceptibility

For the protease susceptibility assay, the H7 HA in the experimental group was firstly mixed with H7.5 Fab with molar ratio of 1:3 and diluted to 2 mg/ml (for H7 only) in the buffer of 20 mM Tris-HCl (pH 8.0) and 150 mM NaCl. The control group H7 was diluted with the same buffer to the sample concentration. Both groups of H7 samples were then aloquated and mixed with 0%, 0.1%, 0.2%, 1%, and 2% of trypsin (dissolved in the same buffer above), and each reaction was incubated at room temperature overnight. The samples were then submitted for sodium dodecyl suflate polyachrylamide gel electrophoresis (SDS-PAGE) analysis to determine the stability of the H7 protein.

### Biolayer inferometry

An Octet RED instrument (FortéBio, Inc.) was used to determine K_d_ of the antibody–antigen interactions by biolayer interferometry. To determine the binding affinity of H7.5 Fab to its ligands, HAs (biotinylated as previously described [3,37]) were immobilized onto streptavidin-coated biosensors (FortéBio, Inc.) and incubated with H7.5 Fab at 62.5–500 nM for 120 seconds for association and then incubated in PBS buffer with 0.2% BSA for dissociate for 120 seconds. The signals for each binding event were measured in real time, and K_d_ values were calculated by fitting to a 1:1 bivalent analyte model. In this case, the K_d_ values were estimated to be less than 10^−3^ nM, as no dissociation was observed.

### nsEM

H7.5 IgG was digested with 4% papain w/w for 4 hours before adding iodoacetamide. The Fab was purified using a protein A column and then added to H7 NY HA with a 3x molar excess of Fab and incubated for 30 minutes at room temperature. The complex was purified using an S200i column (GE Healthcare, Amersham, United Kingdom) and immediately added to 400 mesh copper grids with 2% uranyl formate. Micrographs were taken on a Tecnai Spirit with a TemCam F416 camera using Leginon [39]. Particles were selected using DoG Picker [40] and then stacked and aligned into 2D classes in Appion [41] with MRA/MSA [42]. Particles not representing HA trimers or the complex were removed, and a clean stack was processed in RELION [43].

### Cryo-EM

Sample preparation for uncleaved H7 HA was similar to negative stain. Directly after size exclusion chromatography (SEC) purification, the complex was added at 1 mg/mL to 2/2 gold Quantifoil grids with amphipol and frozen in liquid ethane using a Vitrobot. Cryo sample preparation for cleaved H7 HA with H7.5 Fab does not include a column purification step. Complexes were formed, incubated for one minute at room temperature, and immediately frozen.

### High-resolution image processing

1,324 micrographs were recorded of the uncleaved H7 NY HA/H7.5 Fab complex on a Gatan K2 summit detector mounted on a Titan Krios operating at 300 kV. Data were collected in counting mode at a nominal magnification of 29,000x. Dose rate was approximately 10 electrons/(camera_pixel*s), and frame exposure time was 200 ms. Total exposure time was 10 seconds, with a total dose of 67 electrons/Å^2^. Movie micrograph frames were aligned using MotionCor2 [44], dose weighted, and integrated. Contrast transfer function (CTF) models were determined in GCTF [45]. Projection image candidates of H7 NY HA/H7.5 were identified using a difference-of-Gaussians picker [40]. The resulting set of candidate projection images were subjected to 2D class averaging implemented in RELION 2.1b1 [43]. 227,202 projection images that generated well-formed class averages were selected for further data processing. Iterative angular reconstitution and reconstruction was attempted. Due to on-symmetry axis preferred orientations, reconstruction artefacts were insurmountable for asymmetric refinement. C3 symmetry was imposed in a second attempt, and a stable, albeit somewhat stretched, 3D reconstruction of the data set was obtained. The data set was then subjected to 3D classification, and a subclass of the data (30,032 projection images) was identified; its reconstruction was characterized by persistent Fab densities—indicating full stoichiometric occupancy—and no obvious reconstruction artifacts. This subclass of data was then refined under C3 symmetry constraint to a final resolution of 3.5 Å. Fab Fv regions were well resolved, as were HA1 and part of HA2. The membrane-proximal stem of HA2 was largely disordered. Attempting to recover a structure of the membrane-proximal stem, a globular mask was placed encompassing the disordered part of the map. Inverting the mask, the ordered part of the map could be isolated from which a mask was created to isolate the constant part of the original map. This constant part map was then used to project onto R^2^ along projection directions deduced from Euler and X,Y coordinate assignments obtained during the earlier-referenced refinement, followed by subtraction from the original projection images. This density-subtracted data set was subjected to further iterative angular reconstitution and reconstruction, resulting in a density map of the membrane-proximal stem resolved to 3.9 Å. To investigate if the two maps could be combined and interpreted as one instance of multiple only locally diverging—breathing—conformations, the Euler and X,Y coordinates determined in the stem refinement was applied to the corresponding original projection images and the coinciding 3D object reconstructed. This density map exhibited, albeit noisy, density for the constant part of the structure, suggesting that the membrane-proximal part of the stem is breathing locally around the position determined in the local map. Treatment of our cleaved H7 NL HA/H7.5 and H7 Sh2 HA/H7.5 data sets was performed in RELION 2.1b1 and followed a standard procedure, as outlined above, prior to local refinement procedures and as outlined in the supplemental information (S5 Fig). FSC 0.143 between independently refined half sets were 7.4 Å (H7 Sh2 HA/3 H7.5 Fabs), 9.2 Å (H7 NL HA/3 H7.5 Fabs), and 10.2 Å (H7 NL HA/2 H7.5 Fabs).

### Model building and refinement

A homology model was created (Modeller; [46]) based on the X-ray structure of HA7 HA1/H2 (A/New York/30732-1/2005; PDB 3M5G) [13]. The model was created by combining with the H7.5 Fab X-ray structure and rigid-body fitted to the cryoEM density map. A fragment library consisting of 200 7mers for each amino acid position in the model was compiled from a nonredundant protein structure database. Iterations of manual and Rosetta fragment-library-based centroid rebuilding-and-refinement was then performed [14]. The resulting model was all-atom-refined under constraints of the density map. Glycans were manually built in COOT [34] and final rounds of real-space refinement performed in PHENIX 1.12 [47]. The membrane-proximal part of HA2 was built similarly in the local map referenced above. The builds were then combined into the final structure. Evaluation of builds were performed in MolProbity [35], EMRinger [48], and Privateer [49] and by the PDB validation server.

### HDX-MS

Antigen–antibody complexes were prepared by mixing HA H7 and H7.5 antibodies at 1:1.1 stoichiometric ratio and incubating at room temperature for 30 minutes and then kept at 0 °C. For control experiments, free antigens were also diluted with the same volume of protein buffer and treated the same way as complexes. To initiate HDX reactions, 2 μl of prechilled protein stock solution (free uncleaved HA NL H7, 1.8 mg/ml; H7.5-uncleaved HA NL H7, 4.5 mg/ml; cleaved HA NL H7, 1.6 mg/ml; or H7.5-cleaved HA NL H7, 4.5 mg/ml) was diluted into 4 μl D_2_O buffer (8.3 mM Tris, 150 mM NaCl, in D_2_O, pD_READ_ 7.2) at 0 °C. At the indicated times of 10 seconds, 100 seconds, 1,000 seconds, 10,000 seconds, and 100,000 seconds, the exchange reaction was quenched by the addition of 9 μl of optimized quench solution (6.4 M GuHCl, 1 M TCEP, 0.8% formic acid [pH 2.4]) at 0 °C. After incubating on ice for 5 minutes, the quenched sample was diluted 5-fold with 0.8% formic acid containing 16.6% glycerol, immediately frozen on dry ice, and stored at −80 °C. In addition, undeuterated samples and equilibrium-deuterated control samples were also prepared. All samples were then loaded onto our in-house DXMS apparatus for online digestion and separation. The resulting peptides were directed into an OrbiTrap Elite Mass Spectrometer (Thermo Fisher Scientific, San Jose, California) for DXMS analysis. Instrument settings have been optimized for HDX analysis. The data acquisition was carried out in a data-dependent mode and the 5 or 10 most abundant ions were selected for MS/MS analysis. Proteome Discoverer software was used for peptide identification. The centroids of each peptide was calculated with HDExaminer and then converted to corresponding deuteration levels with corrections for back exchange. The deuteration levels of the peptides were further sublocalized using overlapping peptides by MATLab program.

### Epitope conservation analysis

Nonduplicate HA sequences were fetched from the databases at www.fludb.org in FASTA format. Strain A/Aichi/2/1968 HA gene (Genbank Accession M55059) was then defined as a reference for residue numbering. We then used an in-house script to build a database of every HA sequence’s residues at every position relative to the reference. First, we performed pairwise Clustal alignment of every sequence to the reference sequence [50]. The results were parsed into a database keyed on positions relative to the reference, with gaps notated as subpositions following the last identical residue. We are then able to interrogate the database by providing position numbers and desired residues at the position, providing an output of sequences that meet the search criteria as well as breakdowns of residues at the positions. Analysis of natural HA variants was done, as previously described in Wu and colleagues, 2018 [51]. Briefly, a total of 6,984 HA sequences made up of a subset of up to 20 sequences per year per subtype were analyzed from the Global Initiative for Sharing Avian Influenza Data (GISAID; https://gisaid.org). Sequences were aligned using MAFFT version 7.157b [52]. Sequence logos were generated by WebLogo [53].

## Supporting information

S1 Fig Potent binding of H7.5 Fab to the targeted H7 HAs. The association and disassociation curves from biolayer interferometry of H7.5 Fab to the targeted H7 HAs Shanghai/2/2013 (A) and Netherlands/219/2003 (B) are presented. K_d_ values are estimated to be less than 10^−3^ nM with 1:1 fitting, as no dissociation of the Fab was observed. As previously reported in Thornburg and colleagues [10], the H7.5 antibody shows small amounts of HAI activity against H7 Eurasian and North American H7 strains (C), as well as neutralizing activity against H7N9 Shanghai but not H3N2 Sydney (D). Fab, fragment antigen binding; HA, hemagglutinin.
(TIF)

S2 Fig Raw micrographs and 2D class averages of cleaved HAs and complexes with H7.5 in nsEM. Cleaved H7 unliganded trimers in nsEM (top row). H7 Shanghai cleaved in complex H7.5 Fab at 4 °C at different incubation times (middle row). H7 New York and H7 Netherlands in complex with H7.5 Fab for 5 minutes and 3 hours, respectively, at 4 °C (bottom row). Fab, fragment antigen binding; HA, hemagglutinin; nsEM, negative-stain electron microscopy.
(TIF)

S3 Fig Binding of antibody H7.5 increases the protease susceptibility of H7 HA to trypsin digestion. Purified H7 Sh2 HA trimer was incubated with H7.5 Fab (molar ratio of 1:3) or same volume of buffer. The samples were digested with different percentages of trypsin indicated. SDS-PAGE electrophoresis was used to analyze the H7 Sh2 HA stability. Fab, fragment antigen binding; HA, hemagglutinin.
(TIF)

S4 Fig Protocol for density-subtracted cryoEM data refinement. (A) After data collection, particle extraction, 2D, and 3D classification, all particles resembling the H7 complex were submitted to 3D refinement, resulting in a reconstruction that had diffuse density corresponding to the stem region at the base of the trimer. We therefore subtracted this region from the map and re-refined particles using focused classification. (B) FSC plots of full map and stem base. (C) Resolution maps of full map and stem base show lower resolution in stem alone. (D) Resolution voxel distribution. (E) Relative angular distribution of full map shows bias in side views. cryoEM, cryo-electron microscopy; FSC, Fourier shell correlation.
(TIF)

S5 Fig Local cryoEM data refinement of membrane-proximal part of H7 NY HA0 in complex with H7.5 Fab. (A) Standard cryoEM data refinement (iterative regularized likelihood optimization of suitable Bayesian posterior functions) results in Euler angular and x, y translational coordinate assignment (Φ, Θ, ψ, x, y)_i_ for each molecular projection image i. (B) As density corresponding to the membrane-proximal part of our H7 NY HA0/H7.5 complex was scattered, we proceeded to subtract density corresponding to the well-ordered part of the complex from each of the projection images i. The resulting projection images (i') were then, during subsequent refinement, subjected to coordinate optimization locally around the preassigned coordinates (Φ’, Θ’, ψ’, x’, y’)_I’_. The resulting density map was well ordered in the membrane-proximal region that was previously disordered. (C) We then applied the local refinement coordinates to the original molecular projection images and performed a 3D reconstruction (Φ’, Θ’, ψ’,x’, y’)_I_. The resulting density map was well ordered in the membrane-proximal region. In addition, density corresponding to the membrane-distal part of H7 NY HA0/H7.5 was recovered, albeit at a lower resolution than observed in the original reconstruction in A. This indicates that the disorder in the membrane-proximal part of the complex observed in the original reconstruction was a result of local positional variation (breathing) due to the open base, wherein stabilizing interaction between adjacent protomers is hampered. (D) Same 3D reconstructed map as in C but displayed at lower contour level. Density corresponding to both H7.5 Fab and the membrane-distal part of H7 NY HA0 is clearly visible. cryoEM, cryo-electron microscopy; Fab, fragment antigen binding; HA, hemagglutinin.
(TIF)

S6 Fig Surface 2 in the H7.5-interacting epitope that is proximal to H-FR3 of the antibody. H7.5 binds to adjacent protomers. Zoomed view of binding surface 1 (dark gold) on protomer 1, binding surface 2 (light gold), and RBS (green) of protomer 2. Residues shown as sticks on H-FR3 interact with surface 2 and fit into the neighboring RBS. H-FR3, heavy-chain framework region 3; RBS, receptor-binding site.
(TIF)

S7 Fig Sequence logos of H7.5 epitope across different influenza subtypes. Analysis of the targeted epitope of H7.5 antibody from HA sequences, including avian and other zoonotic strains, shows a single conserved proline at 62, except H8, which shows no sequence similarity to H7 in the H7.5 epitope. HA, hemagglutinin.
(TIF)

S8 Fig HDX-MS data. (A) Blue suggests the HA regions that exchange slower upon H7.5 Ab binding, and red suggests regions that exchange faster upon binding. (B) Blue suggests the regions that exchange slower upon H7.5 Ab binding, and red suggests the HA regions that exchange faster upon binding. (C) Blue suggests the HA regions that exchange slower in cleaved HA H7, and red suggests the HA regions that exchange faster in cleaved HA H7. HDX profiles for uncleaved (D) and cleaved (E) HA as a function of time. These analyses indicated regions in the respective forms of HA that are more or less accessible to exchange. The exchange profiles for the regions resolved are overall very similar. HA, hemagglutinin; HDX-MS, hydrogen–deuterium exchange mass spectrometry.
(TIF)

S9 Fig Processing cryoEM data of cleaved HA. (A) Data processing and refinement of H7 Shanghai and H7.5 Fab. (B) Data processing and refinement of H7 Netherlands with three and two H7.5 Fabs bound along with angular distribution. (C) FSC plot of H7 Shanghai and H7 Netherlands bound by two or three H7.5 Fabs. cryoEM, cryo-electron microscopy; Fab, fragment antigen binding; FSC, Fourier shell correlation; HA, hemagglutinin.
(TIF)

S10 Fig Comparison of head-splitting antibodies. Crystal structure of m826 (PDB 5VAG) bound to H1 head was aligned to the H1 head region of our cleaved H7 NY model to compare the epitope with H7.5. There is no overlap between antibodies. PDB, Protein Data Bank.
(TIF)

## Abbreviations

bnAb: broadly neutralizing antibody
CDRH3: complementary determining region heavy chain 3
cryoEM: cryo-electron microscopy
Fab: fragment antigen binding
FSC: Fourier shell correlation
HA: hemagglutinin
HDX-MS: hydrogen–deuterium exchange mass spectrometry
H-FR3: heavy-chain framework region 3
mAb: monoclonal antibody
NA: neuraminidase
nsEM: negative-stain electron microscopy
PDB: Protein Data Bank
RBS: receptor-binding site
smFRET: single molecule Förster resonance energy transfer
