# Supplementary figures and images for: Potent anti-influenza H7 human monoclonal antibody induces separation of hemagglutinin receptor-binding head domains
Source: PLoS Biol. 2019 Feb 4;17(2):e3000139. doi: 10.1371/journal.pbio.3000139 (PMC6375650; doi:10.1371/journal.pbio.3000139)

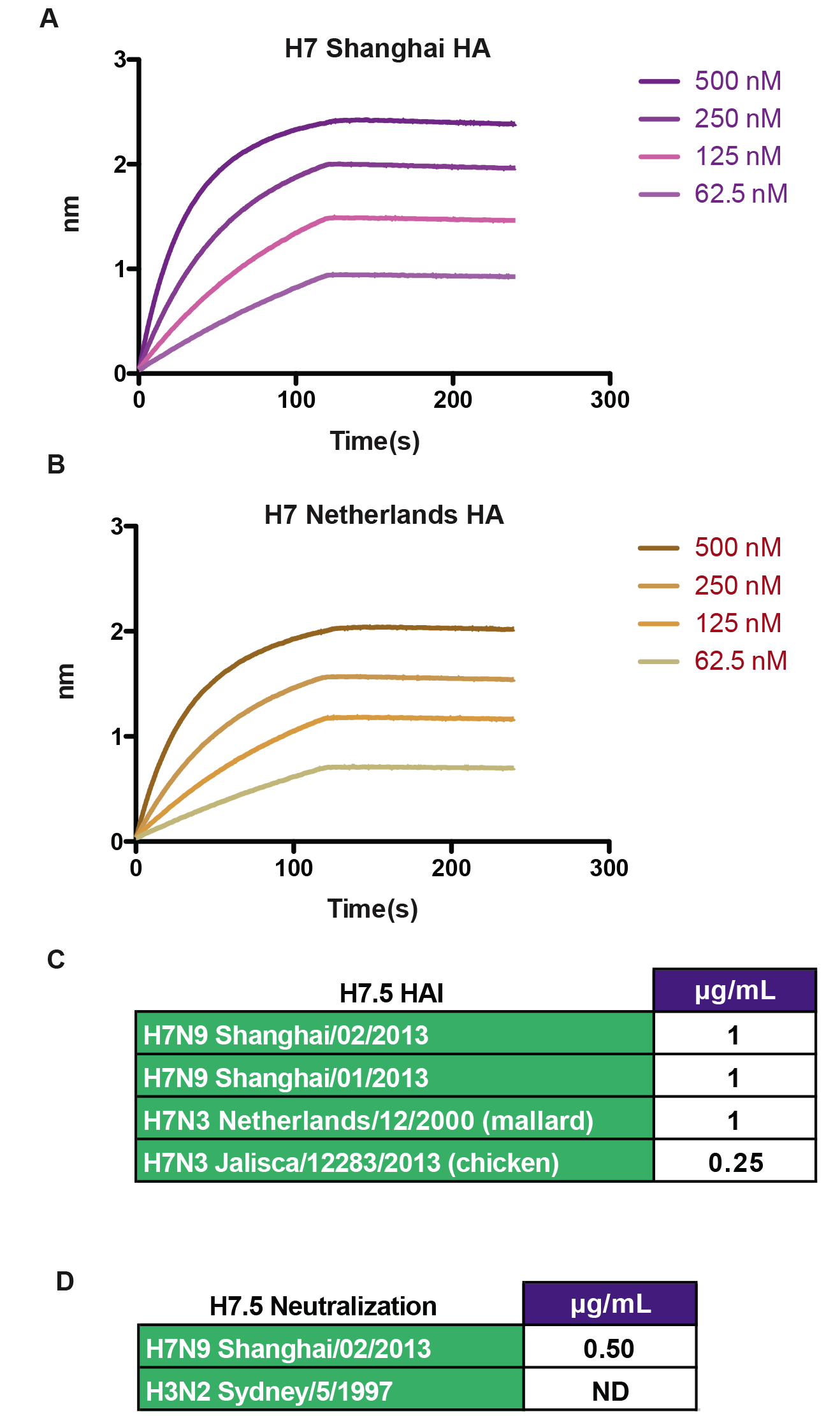

Supplement: S1 Fig — The association and disassociation curves from biolayer interferometry of H7.5 Fab to the targeted H7 HAs Shanghai/2/2013 (A) and Netherlands/219/2003 (B) are presented. Kd values are estimated to be less than 10−3 nM with 1:1 fitting, as no dissociation of the Fab was observed. As previously reported in Thornburg and colleagues [10], the H7.5 antibody shows small amounts of HAI activity against H7 Eurasian and North American H7 strains (C), as well as neutralizing activity against H7N9 Shanghai but not H3N2 Sydney (D). Fab, fragment antigen binding; HA, hemagglutinin. (TIF) [file pbio.3000139.s001.tif]

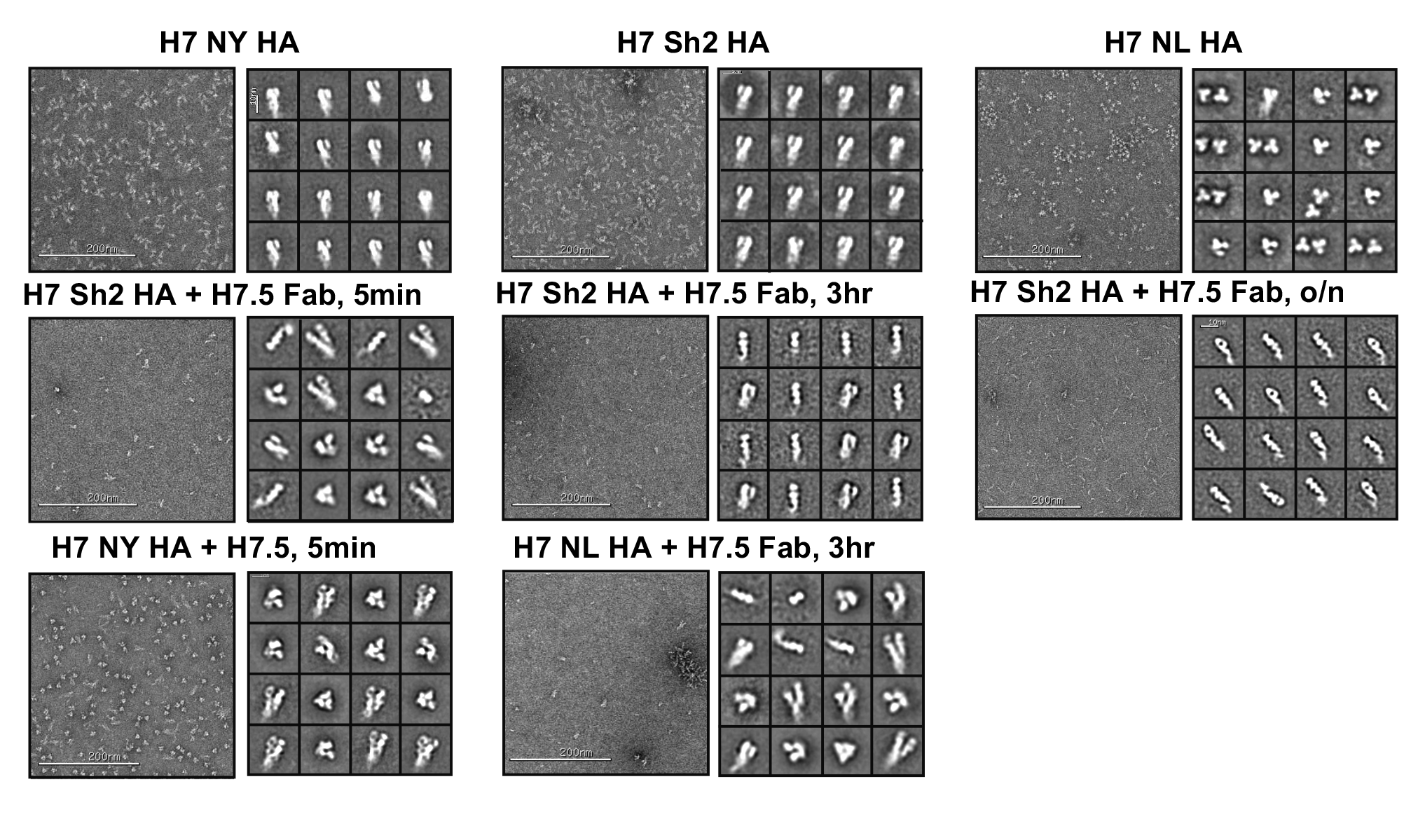

Supplement: S2 Fig — Cleaved H7 unliganded trimers in nsEM (top row). H7 Shanghai cleaved in complex H7.5 Fab at 4 °C at different incubation times (middle row). H7 New York and H7 Netherlands in complex with H7.5 Fab for 5 minutes and 3 hours, respectively, at 4 °C (bottom row). Fab, fragment antigen binding; HA, hemagglutinin; nsEM, negative-stain electron microscopy. (TIF) [file pbio.3000139.s002.tif]

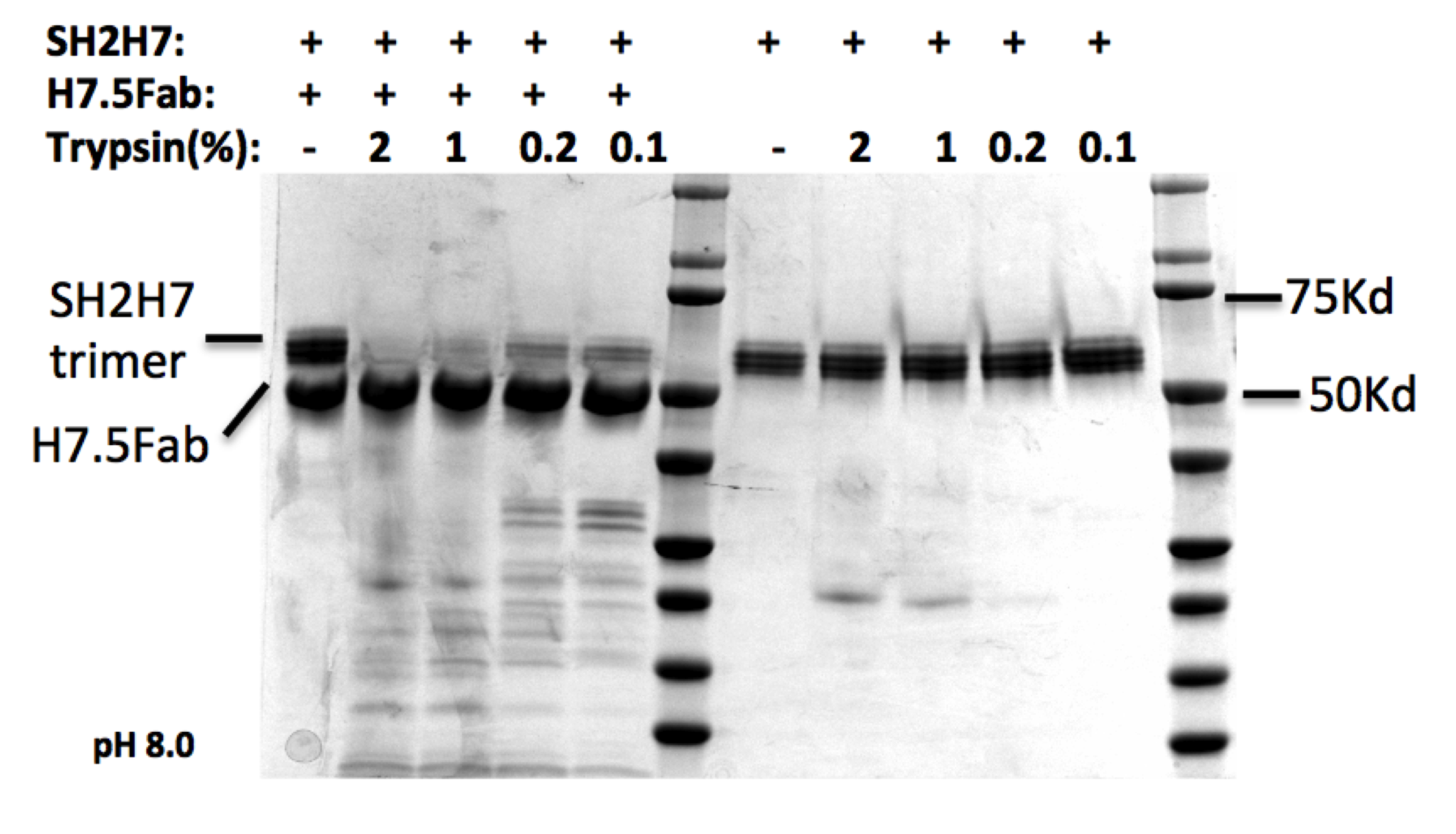

Supplement: S3 Fig — Purified H7 Sh2 HA trimer was incubated with H7.5 Fab (molar ratio of 1:3) or same volume of buffer. The samples were digested with different percentages of trypsin indicated. SDS-PAGE electrophoresis was used to analyze the H7 Sh2 HA stability. Fab, fragment antigen binding; HA, hemagglutinin. (TIF) [file pbio.3000139.s003.tif]

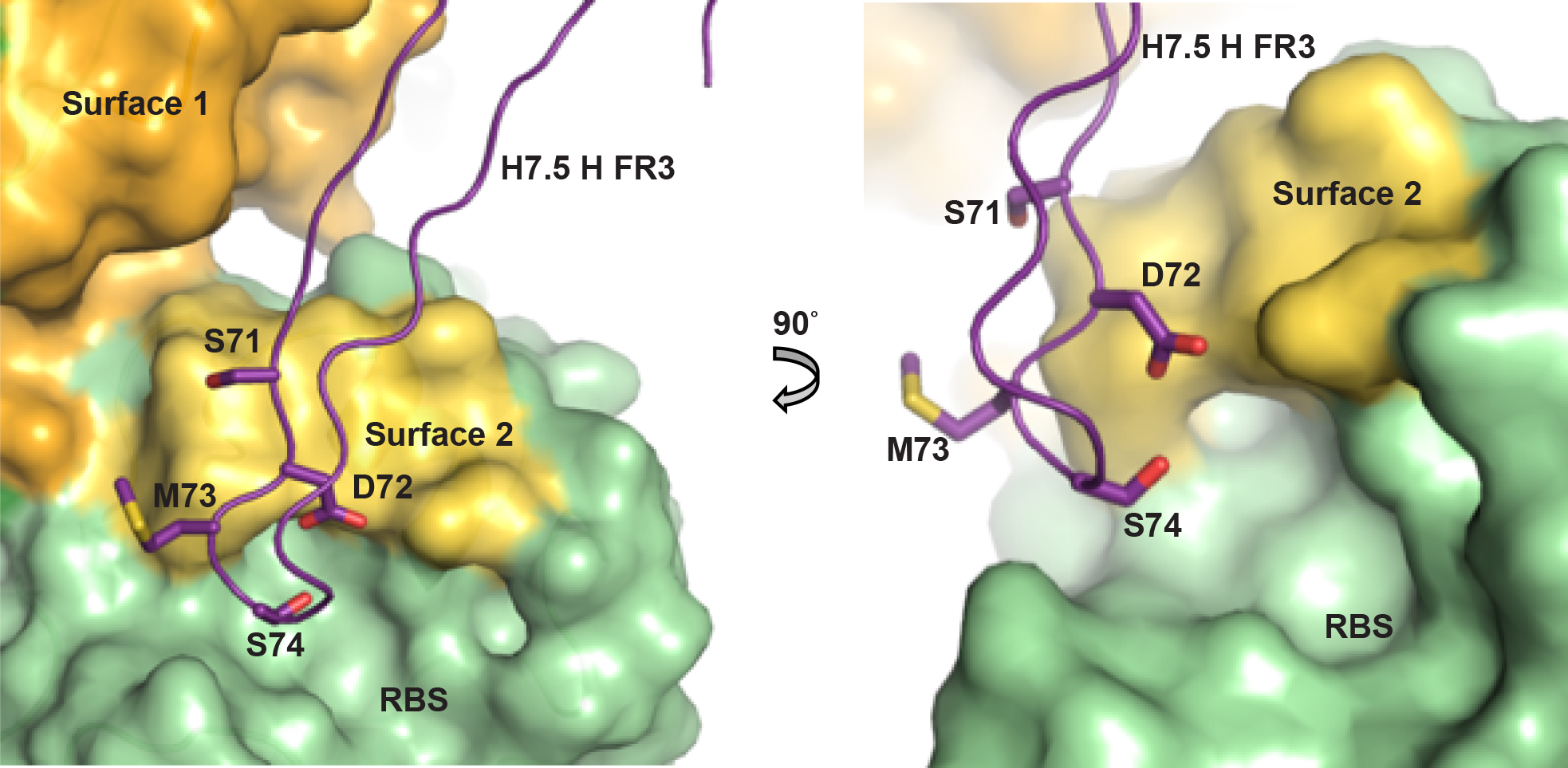

Supplement: S4 Fig — (A) After data collection, particle extraction, 2D, and 3D classification, all particles resembling the H7 complex were submitted to 3D refinement, resulting in a reconstruction that had diffuse density corresponding to the stem region at the base of the trimer. We therefore subtracted this region from the map and re-refined particles using focused classification. (B) FSC plots of full map and stem base. (C) Resolution maps of full map and stem base show lower resolution in stem alone. (D) Resolution voxel distribution. (E) Relative angular distribution of full map shows bias in side views. cryoEM, cryo-electron microscopy; FSC, Fourier shell correlation. (TIF) [file pbio.3000139.s004.tif]

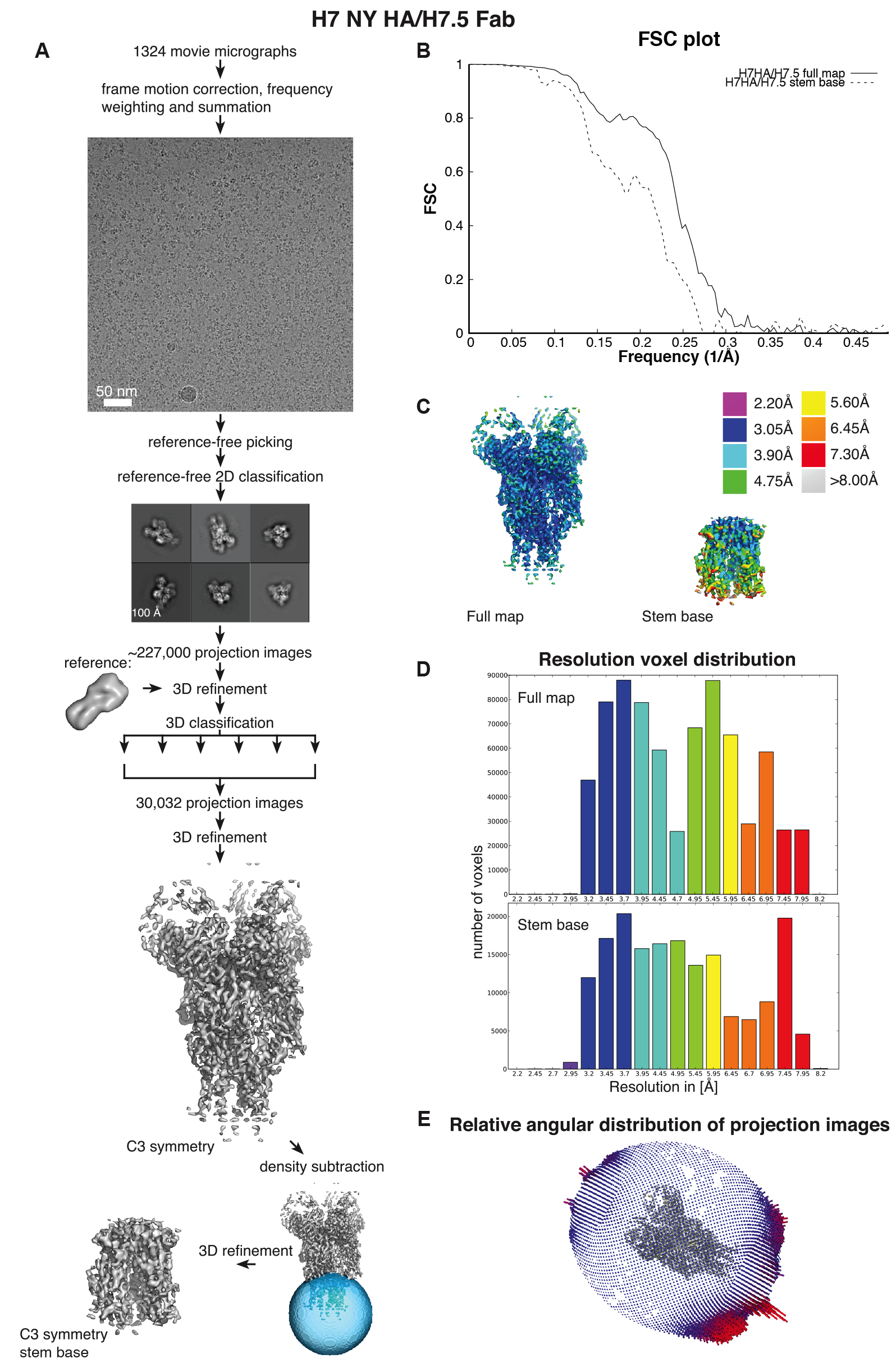

Supplement: S5 Fig — (A) Standard cryoEM data refinement (iterative regularized likelihood optimization of suitable Bayesian posterior functions) results in Euler angular and x, y translational coordinate assignment (Φ, Θ, ψ, x, y)i for each molecular projection image i. (B) As density corresponding to the membrane-proximal part of our H7 NY HA0/H7.5 complex was scattered, we proceeded to subtract density corresponding to the well-ordered part of the complex from each of the projection images i. The resulting projection images (i') were then, during subsequent refinement, subjected to coordinate optimization locally around the preassigned coordinates (Φ’, Θ’, ψ’, x’, y’)I’. The resulting density map was well ordered in the membrane-proximal region that was previously disordered. (C) We then applied the local refinement coordinates to the original molecular projection images and performed a 3D reconstruction (Φ’, Θ’, ψ’,x’, y’)I. The resulting density map was well ordered in the membrane-proximal region. In addition, density corresponding to the membrane-distal part of H7 NY HA0/H7.5 was recovered, albeit at a lower resolution than observed in the original reconstruction in A. This indicates that the disorder in the membrane-proximal part of the complex observed in the original reconstruction was a result of local positional variation (breathing) due to the open base, wherein stabilizing interaction between adjacent protomers is hampered. (D) Same 3D reconstructed map as in C but displayed at lower contour level. Density corresponding to both H7.5 Fab and the membrane-distal part of H7 NY HA0 is clearly visible. cryoEM, cryo-electron microscopy; Fab, fragment antigen binding; HA, hemagglutinin. (TIF) [file pbio.3000139.s005.tif]

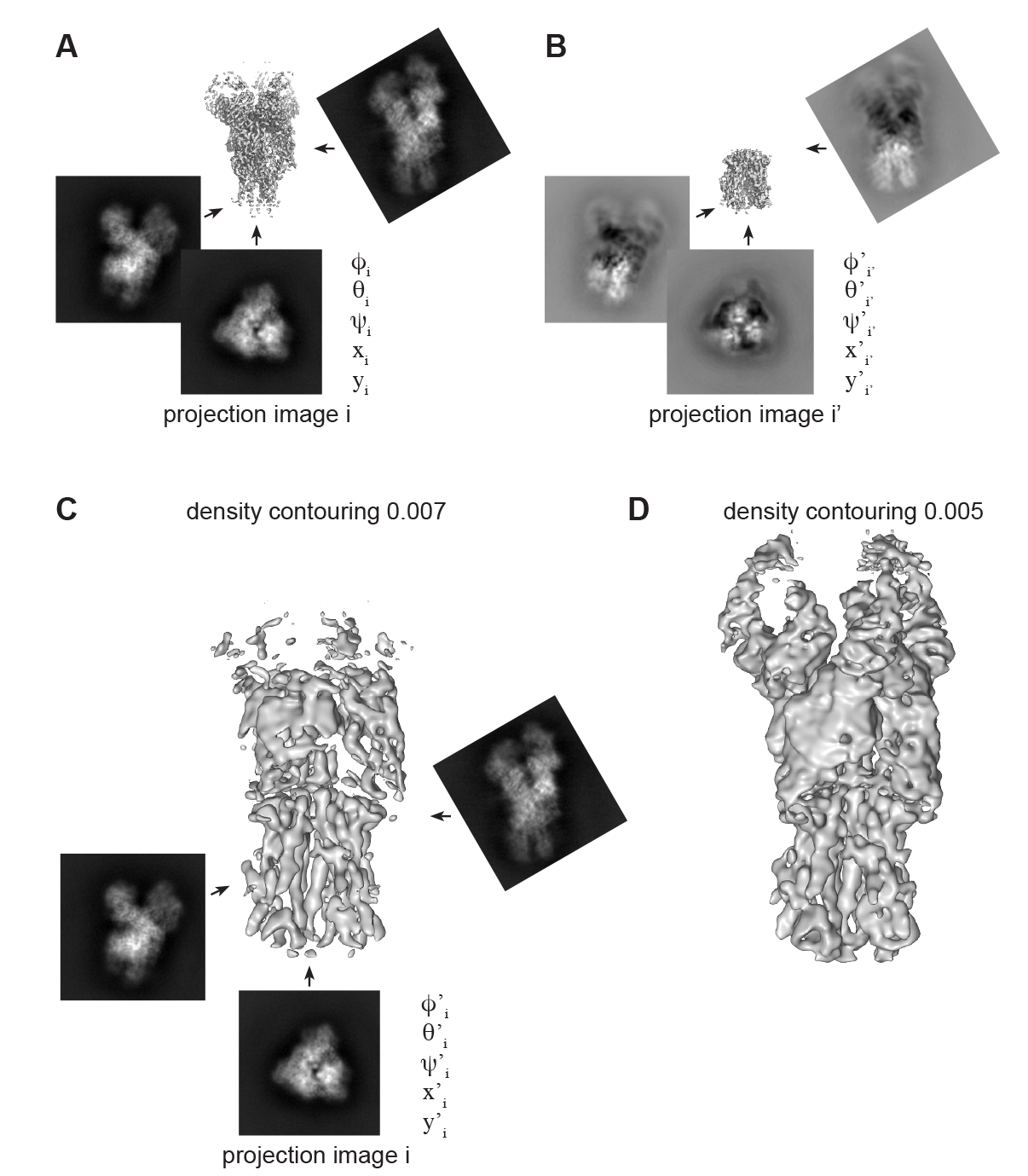

Supplement: S6 Fig — H7.5 binds to adjacent protomers. Zoomed view of binding surface 1 (dark gold) on protomer 1, binding surface 2 (light gold), and RBS (green) of protomer 2. Residues shown as sticks on H-FR3 interact with surface 2 and fit into the neighboring RBS. H-FR3, heavy-chain framework region 3; RBS, receptor-binding site. (TIF) [file pbio.3000139.s006.tif]

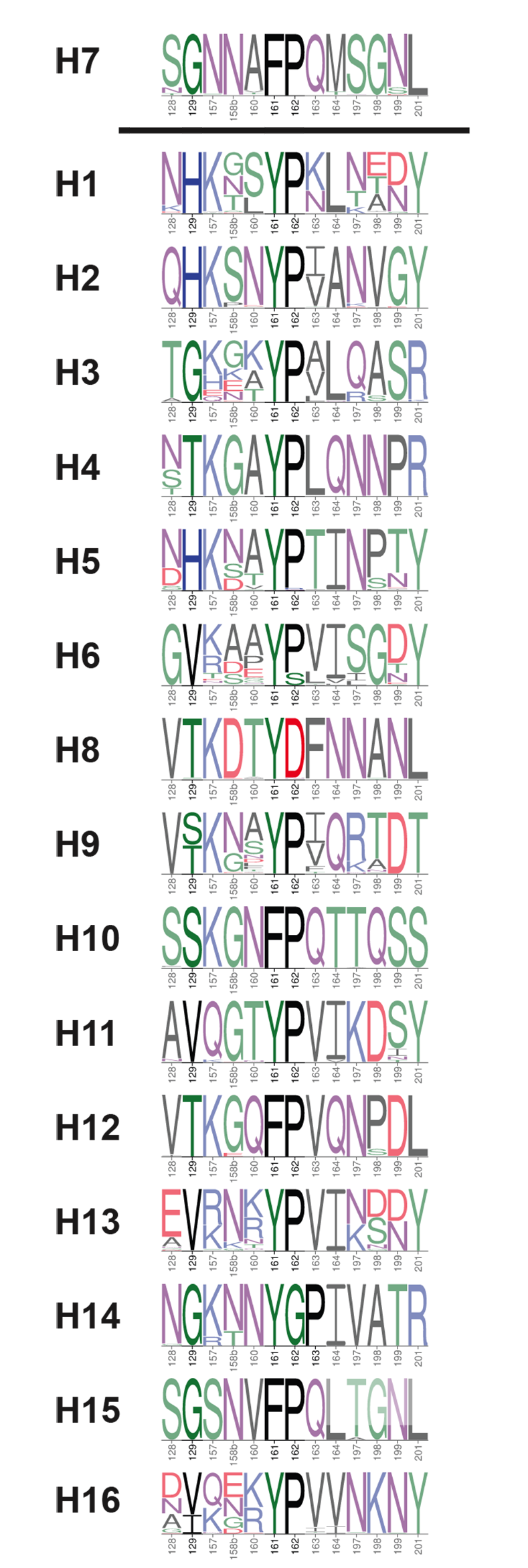

Supplement: S7 Fig — Analysis of the targeted epitope of H7.5 antibody from HA sequences, including avian and other zoonotic strains, shows a single conserved proline at 62, except H8, which shows no sequence similarity to H7 in the H7.5 epitope. HA, hemagglutinin. (TIF) [file pbio.3000139.s007.tif]

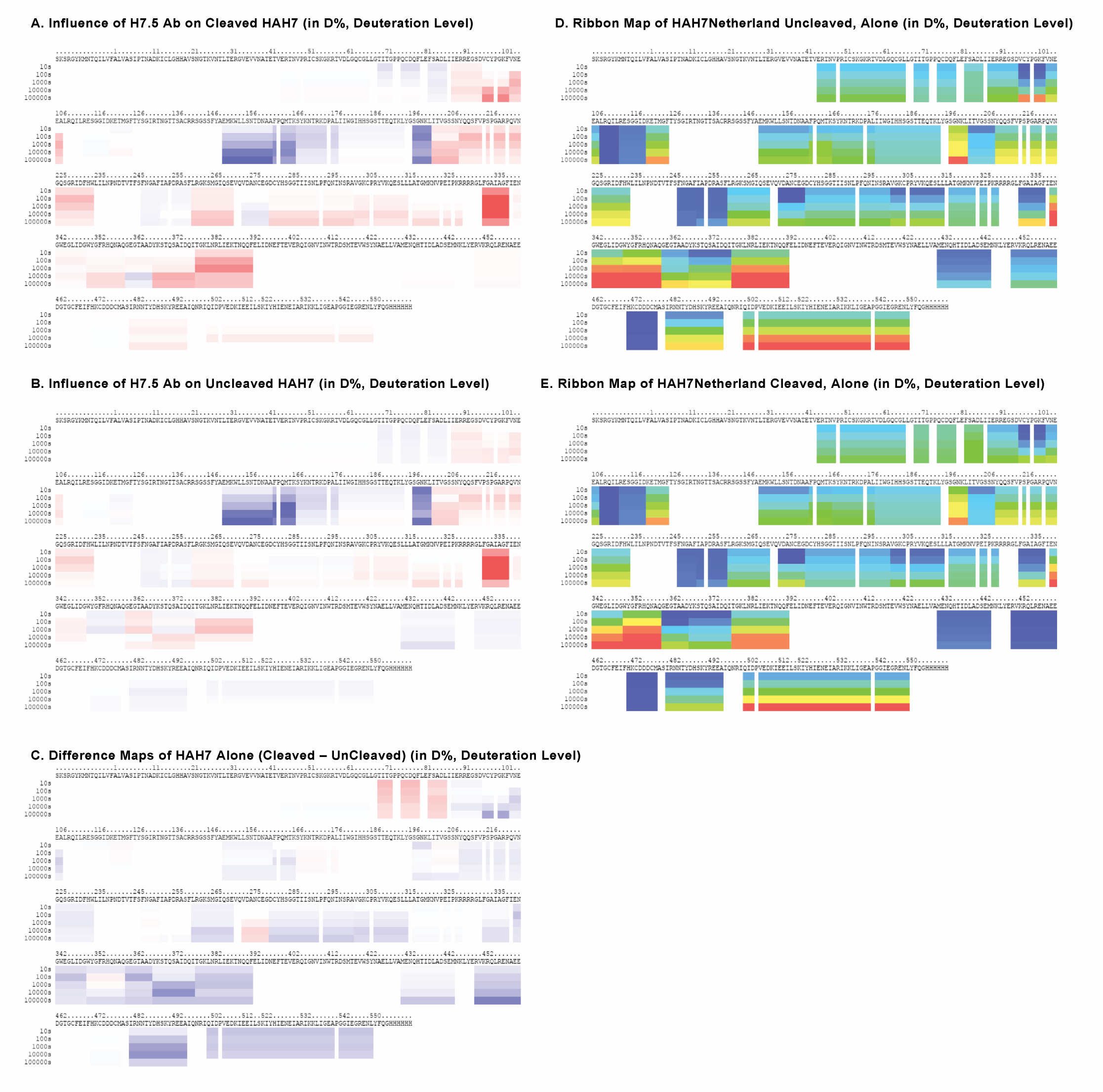

Supplement: S8 Fig — (A) Blue suggests the HA regions that exchange slower upon H7.5 Ab binding, and red suggests regions that exchange faster upon binding. (B) Blue suggests the regions that exchange slower upon H7.5 Ab binding, and red suggests the HA regions that exchange faster upon binding. (C) Blue suggests the HA regions that exchange slower in cleaved HA H7, and red suggests the HA regions that exchange faster in cleaved HA H7. HDX profiles for uncleaved (D) and cleaved (E) HA as a function of time. These analyses indicated regions in the respective forms of HA that are more or less accessible to exchange. The exchange profiles for the regions resolved are overall very similar. HA, hemagglutinin; HDX-MS, hydrogen–deuterium exchange mass spectrometry. (TIF) [file pbio.3000139.s008.tif]

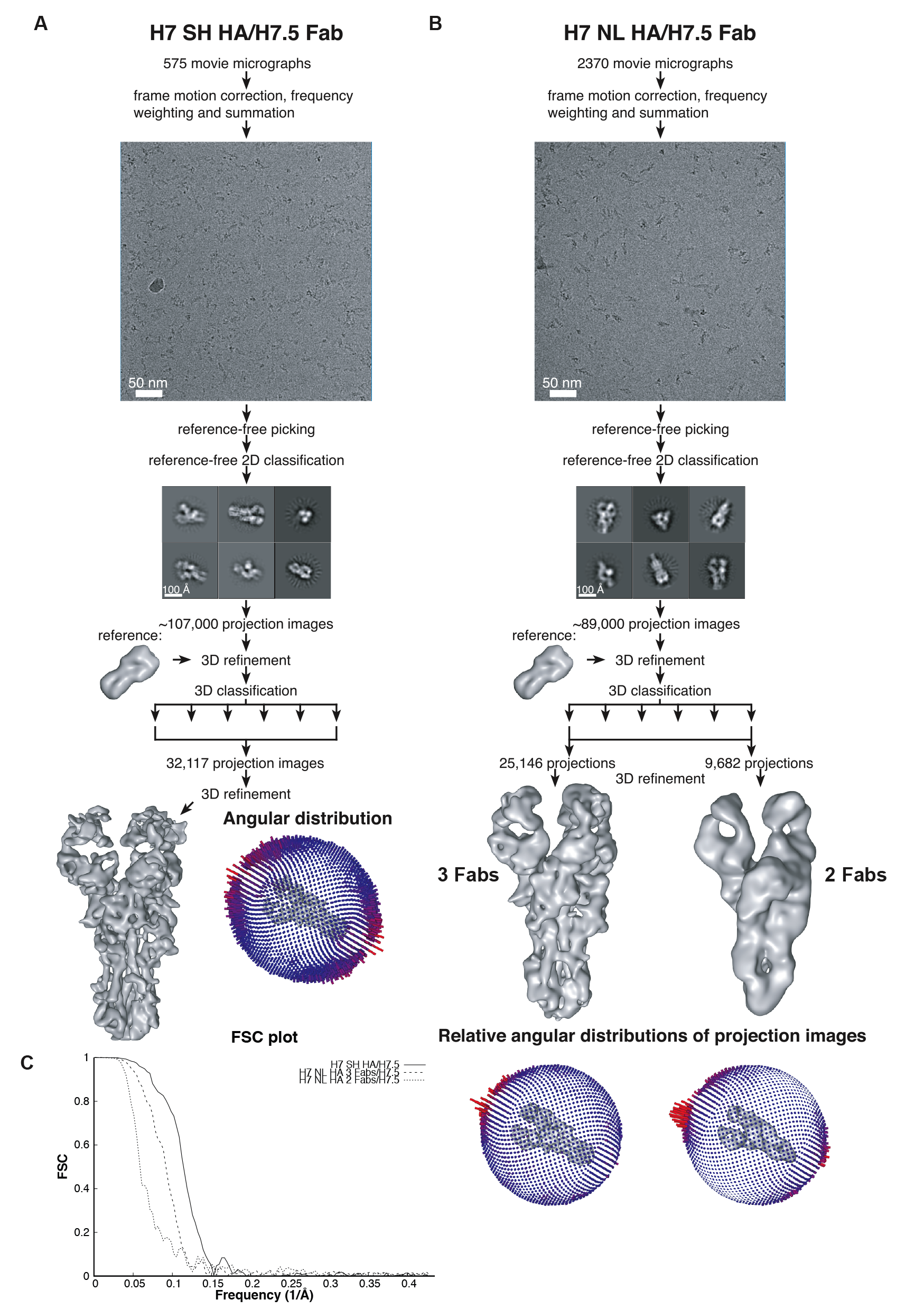

Supplement: S9 Fig — (A) Data processing and refinement of H7 Shanghai and H7.5 Fab. (B) Data processing and refinement of H7 Netherlands with three and two H7.5 Fabs bound along with angular distribution. (C) FSC plot of H7 Shanghai and H7 Netherlands bound by two or three H7.5 Fabs. cryoEM, cryo-electron microscopy; Fab, fragment antigen binding; FSC, Fourier shell correlation; HA, hemagglutinin. (TIF) [file pbio.3000139.s009.tif]

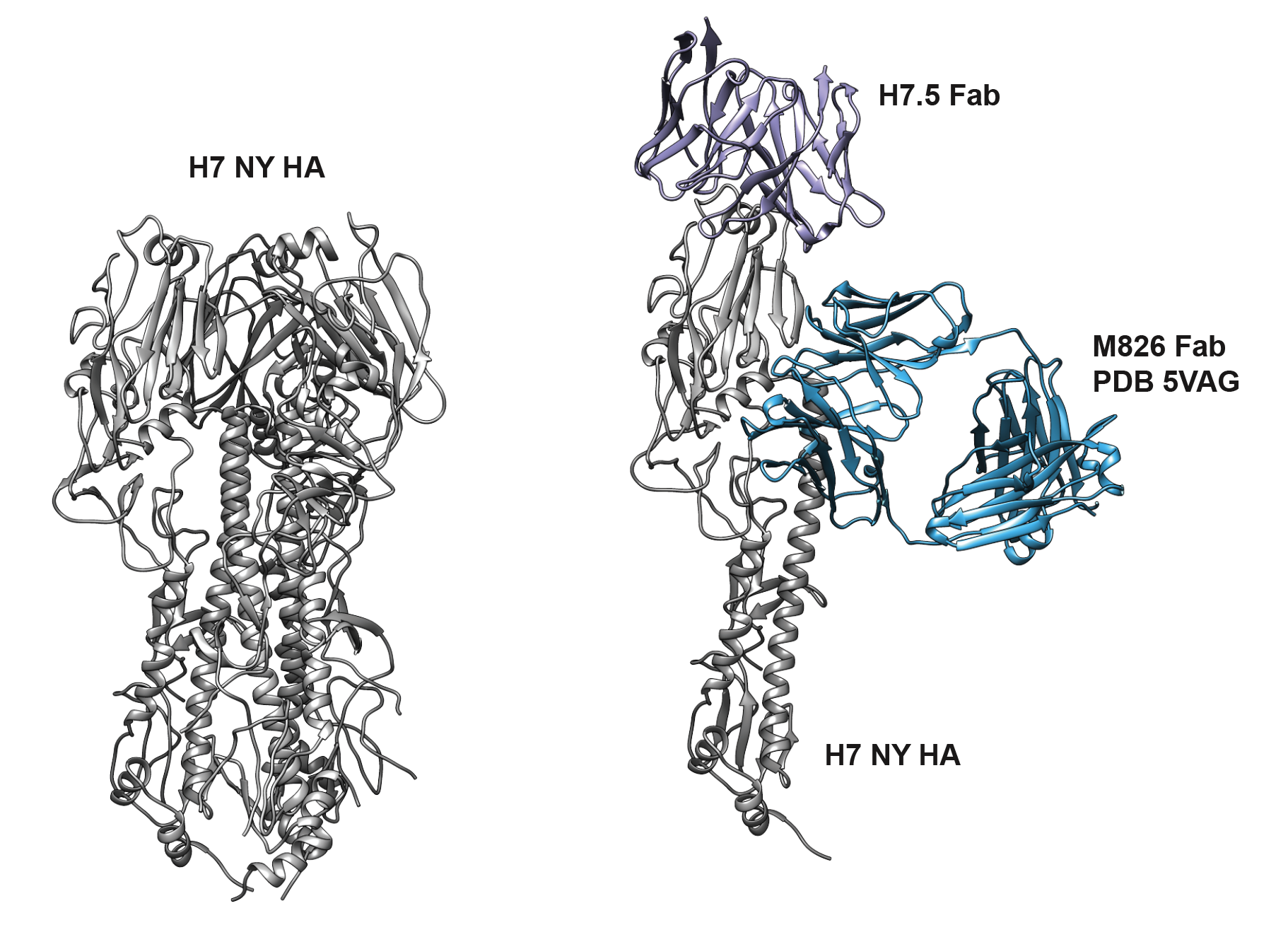

Supplement: S10 Fig — Crystal structure of m826 (PDB 5VAG) bound to H1 head was aligned to the H1 head region of our cleaved H7 NY model to compare the epitope with H7.5. There is no overlap between antibodies. PDB, Protein Data Bank. (TIF) [file pbio.3000139.s010.tif]
